# Perovskite‐Based Catalysts for the Oxygen Evolution Reaction: Synthesis, Device Relevant Performance, and Scale‐Up Strategies

**DOI:** 10.1002/smll.75231

**Published:** 2026-08-22

**Authors:** Jala Bib Khan, Norbert Kazamer, Marco Brand, Tim Hülser, Clemens Pollerberg, Ulrich Rost

**Affiliations:** ^1^ Westphalian Energy Institute Westfälische Hochschule University of Applied Sciences Gelsenkirchen Germany; ^2^ ProPuls GmbH Gelsenkirchen Germany; ^3^ Politehnica University Timișoara, Department of Materials Engineering and Manufacturing Timișoara Romania; ^4^ Institut für Umwelt & Energie Technik & Analytik e. V. (IUTA) Duisburg Germany; ^5^ Center for Nanointegration Duisburg‐Essen (CENIDE) Duisburg Germany; ^6^ Hochschule RheinMain University of Applied Sciences and Arts Department of Engineering, Am Brückweg Rüsselsheim am Main Germany

**Keywords:** catalytic activity, in situ characterization, oxygen evolution reaction, perovskite catalysts, proton exchange membrane, scale‐up strategies

## Abstract

Perovskite‐based oxides are promising electrocatalysts for the oxygen evolution reaction due to their flexible composition, tunable electronic structure, and robust nature. However, their activity and durability in proton exchange membrane (PEM)–relevant acidic conditions are not fully understood, limiting their use in water electrolyzers. This review critically discusses recent advances in perovskite‐based electrocatalysts, covering design, synthesis strategies, morphological engineering, reaction mechanisms, and degradation pathways. Fundamental design principles based on A‐/B‐site engineering, defect chemistry, metal–oxygen covalency, active‐site optimization, and controlled surface reconstruction are discussed together with emerging oxide pathway mechanisms. Particular emphasis is placed on bridging fundamental electrochemical studies with practical implementation through membrane electrode assembly design, catalyst‐layer optimization, in situ evaluation, and realistic performance benchmarking. Finally, current challenges associated with standardization, scalability, and industrial implementation are highlighted, and future research directions for developing durable, low‐PGM perovskite electrocatalysts for PEM water electrolysis are outlined.

## Graphical abstract

Perovskites are emerging as catalysts for acidic oxygen evolution in proton exchange membrane water electrolyzers. This manuscript focuses on catalyst behavior under device‐relevant conditions, highlighting surface reconstruction, elemental utilization, and membrane electrode assembly performance. By linking materials design with electrolyzer operation, it outlines pathways toward durable, low‐PGM anodes for high‐current PEM water electrolysis.

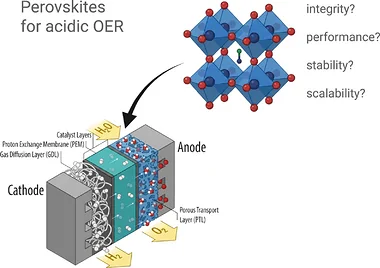

## 1 Introduction

The global transition toward sustainable energy systems has positioned water electrolysis as a key technology for producing green hydrogen, a clean, storable energy carrier with the potential to decarbonize industries ranging from mobility to chemical manufacturing [1, 2, 3]. Water electrolysis is widely categorized into four main types: alkaline water electrolyzers, proton exchange membrane water electrolyzers (PEMWE), anion exchange membrane water electrolyzers, and oxygen ion‐conducting solid oxide electrolyzers. PEMWE has several advantages, including a small system architecture, elevated current density (commercially 1–3 A cm^−2^), rapid start/stop capabilities, high pure hydrogen generation (≥99.99%), and environmental sustainability. These attributes make PEMWEs especially compatible with intermittent renewable energy sources [4, 5].

Central to this process is the oxygen evolution reaction (OER) at the anode, a four‐electron transfer step (Figure 1) that remains a major efficiency bottleneck due to its inherently slow kinetics and high overpotential requirements [6, 7, 8, 9]. In proton exchange membrane (PEM) electrolyzers, where OER proceeds in highly acidic environments, catalysts must endure extreme conditions: low pH, high anodic potentials, and elevated temperatures often exceeding 80°C [5, 10, 11, 12, 13]. These operational demands impose stringent material constraints, particularly in terms of corrosion resistance, electrical conductivity, and long‐term structural integrity [14, 15, 16].

**FIGURE 1:**
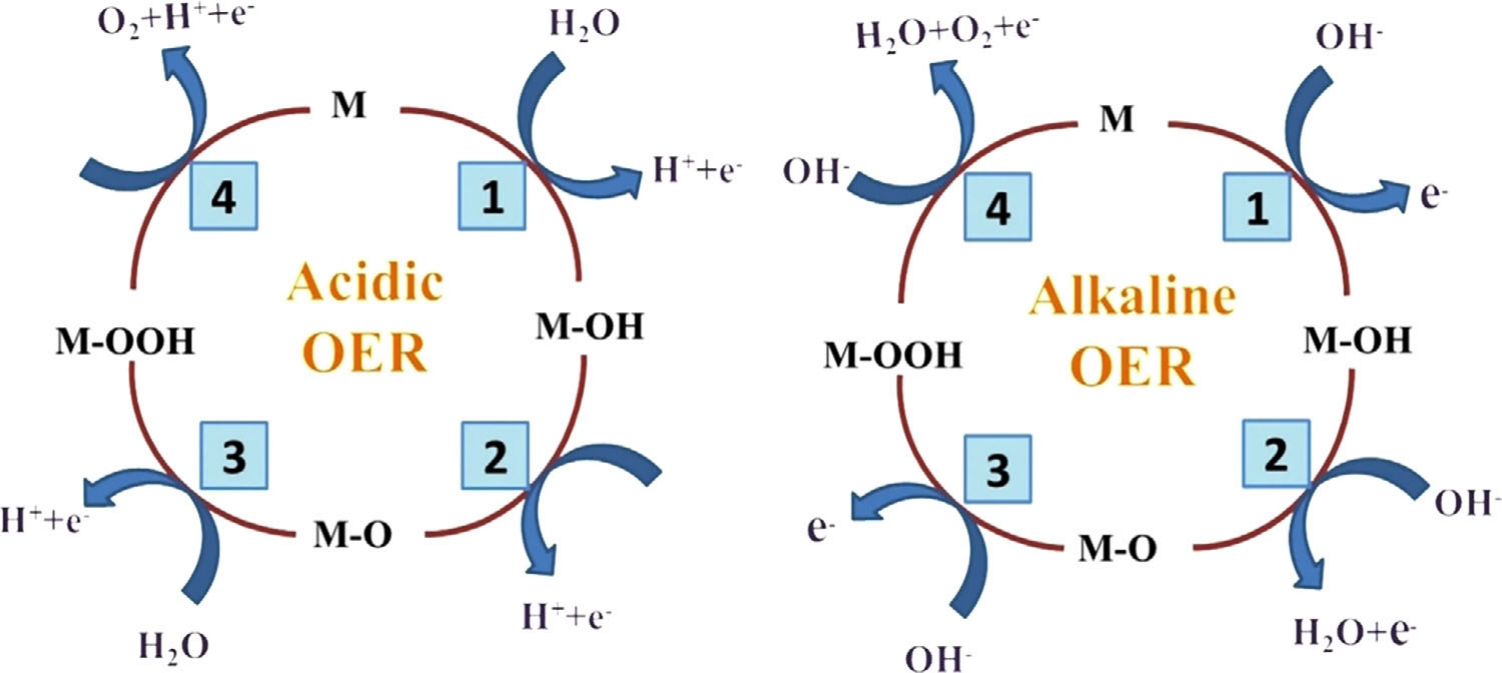
The OER follows a four‐step mechanistic pathway involving four proton‐coupled electron transfer steps in both acidic and alkaline electrolytes. Reproduced with permission [8]. Copyright 2021, John Wiley and Sons.

The thermodynamic potential for water electrolysis under standard conditions is 1.23 V, referred to as the equilibrium potential (*E* ^θ^). In real applications, the capacity to induce water breakdown surpasses the thermodynamic potential. The operational potential of commercial electrolyzers often exceeds 1.23 V [1, 17], indicating a greater electricity need to surmount the inherent activation energy barriers of the anode (*q_a_*) and cathode (*q_c_*), in addition to extra energy losses (*q_other_*) resulting from system resistance [18, 19]. The following equation could articulate the operating voltage (*E_OP_*) of water electrolysis:

$$E_{OP}=1.23+q_{a}+q_{c}+q_{other}$$

To date, iridium, ruthenium, and its oxides remain the most effective electrocatalysts for OER in acidic environments. However, their prohibitive cost and limited global reserves present bottlenecks to large‐scale deployment [13, 20, 21, 22, 23]. This challenge has catalyzed growing interest in alternative structures like the perovskite‐based oxides, a versatile class of materials with the general formula ABO_3_, known for their tunability, lattice stability, and rich redox chemistry [24, 25, 26]. One of the most effective strategies to optimize their physiochemical and electrochemical properties is the intentional substitution of cations with different ionic radii and oxidation states at either crystallographic site. While the A‐site cation primarily governs structural stability, lattice distortion, and oxygen‐ion mobility, the B‐site transition‐metal cation largely determines the electronic structure and redox properties of the perovskite. Consequently, modulation of the B‐site has emerged as one of the most powerful approaches to optimize catalytic activity by controlling the oxidation state, orbital occupancy, adsorption energies of OER intermediates, and the formation of oxygen vacancies [27, 28]. In perovskite‐based electrocatalysts, the catalytic performance is governed by the nature and distribution of the active surface sites, where the adsorption and conversion of reaction intermediates occur. According to the Sabatier principle, optimal activity is achieved when the interaction between these intermediates and the catalyst surface is balanced, avoiding both excessively weak and overly strong adsorption [29, 30]. Since the local atomic and electronic environments vary across the catalyst surface, different sites exhibit different catalytic activities. For acidic OER perovskites, two principal types of active structures have been proposed [31]. In some materials, the crystalline B‐site metal–oxygen octahedral framework remains largely preserved during operation, and the catalytic performance is closely linked to the distortion and connectivity of the BO_6_ octahedra. In others, electrochemical operation induces substantial surface reconstruction, leading to the formation of amorphous B‐site metal–oxygen phases that constitute the catalytically active surface. Variants such as Ruddlesden‐Popper phases, double perovskites, Lanthanum Strontium Manganite (LSM), doped compositions and unconventional Ir‐based perovskites have demonstrated strong promise in reducing/eliminating noble metal content while maintaining competitive OER performance [32, 33, 34, 35, 36, 37]. Strategic surface engineering, such as composite formation with TiO_2_, defect creation, heteroatom doping, interface modulation, and post‐synthesis activation treatments, further enhances their intrinsic activity and durability under PEM‐relevant conditions [38, 39, 40, 41].

Several recent reviews have comprehensively summarized the structural chemistry, reaction mechanisms, defect engineering, and compositional optimization of perovskite oxides, including A‐ and B‐site engineering, heteroatom doping, electronic descriptors, and catalytic mechanisms for the OER [26, 39, 41, 42]. In addition, numerous reviews have focused on the application of perovskite materials in related energy technologies, such as supercapacitors, solar cells, and solid oxide fuel cells, where perovskites are already well‐established functional materials [33, 43]. While these reviews provide valuable insights into the fundamental design and multifunctional properties of perovskite oxides, they generally emphasize intrinsic material properties and laboratory‐scale electrochemical evaluation. In contrast, the present review adopts a device‐oriented perspective by critically assessing acidic perovskite electrocatalysts from fundamental catalyst development to practical implementation. Particular emphasis is placed on synthesis routes suitable for scale‐up, catalyst integration into catalyst layers and membrane electrode assemblies, durability under PEM‐relevant operating conditions, and the transition from ex situ three‐electrode measurements to in situ electrolyzer performance. Rather than providing another comprehensive overview of perovskite materials, this review aims to identify which catalyst concepts successfully translate into practical PEM water electrolysis and to highlight the remaining challenges for industrial implementation.

## 2 Requirements for OER Electrocatalysis in Acidic Environment

Acidic water electrolysis offers several advantages over alkaline systems, including higher voltage efficiency, compact system design, and superior gas purity. However, these benefits come at the cost of significantly harsher operating conditions for anodic catalysts, resulting in limited material choices, reduced durability, and elevated system costs [44]. From a mechanistic perspective, the oxygen evolution reaction (OER) in acidic media involves a sluggish four‐electron transfer process, requiring catalysts that can effectively lower kinetic barriers while withstanding aggressive electrochemical environments [8]. An effective acidic OER catalyst must therefore simultaneously facilitate the adsorption and conversion of oxygen intermediates (*OH, *O, *OOH) and resist oxidative corrosion under sustained anodic polarization [21].

Chemical stability and corrosion resistance constitute the most stringent constraints under acidic conditions. Many transition‐metal oxides that perform well in alkaline media suffer rapid degradation in acid due to high potentials that lead to for example, leaching, lattice destabilization, or phase separation. To date, only a narrow class of oxides—predominantly those containing noble metals such as iridium and ruthenium—exhibit sufficient resilience, although even these materials undergo gradual dissolution and structural reconstruction at high anodic potentials [22, 45, 46]. These observations underscore that intrinsic activity alone is insufficient; long‐term structural integrity under dynamic operating conditions is equally critical.

High electronic conductivity is another indispensable requirement for acidic OER catalysis. Efficient electron transport from active sites to the external circuit is necessary to sustain rapid redox cycling and prevent local charge accumulation that can accelerate catalyst degradation. In this context, perovskite oxides exhibiting metallic or semi‐metallic conductivity are particularly attractive, as they enable continuous electron flow even under high current densities [47].

Operational temperature further exacerbates stability demands. Proton exchange membrane water electrolyzers (PEMWEs) typically operate at temperatures approaching or exceeding 80°C, intensifying chemical corrosion and mechanical stress. Thermal expansion mismatch between the catalyst layer, membrane, and porous transport layers can induce interfacial resistance, delamination, or mechanical failure, necessitating catalysts that are both thermodynamically stable and mechanically compatible with other cell components [48, 49].

Beyond performance and durability, economic considerations impose additional constraints. Despite their outstanding activity and stability, noble‐metal‐based catalysts face severe limitations due to high cost and limited global availability. Consequently, future acidic OER catalysts must minimize noble‐metal loading or partially substitute noble metals with more abundant elements, without compromising durability under PEM‐relevant conditions [50, 51].

Importantly, these requirements—activity, corrosion resistance, conductivity, thermal stability, and cost—are not independent but strongly interrelated. Optimizing one parameter often adversely affects another, highlighting the need for multifunctional catalyst platforms capable of balancing competing demands. Perovskite oxides, with their compositional flexibility, tunable electronic structure, and capacity for controlled surface reconstruction, are therefore uniquely positioned to address these coupled challenges, motivating their detailed examination in the following sections.

## 3 The Foundations for Perovskite Oxides for Acidic OER

### 3.1 Basic Concepts and Mechanistic Pathways

Perovskite oxides, typically described by the general formula ABO_3_, constitute a versatile class of materials for oxygen evolution electrocatalysis owing to their compositional flexibility, structural robustness, and tunable electronic properties. In the perovskite lattice, large rare‐earth or alkaline‐earth cations occupy the A‐site, while transition metal cations reside at the B‐site, forming a network of corner‐sharing BO_6_ octahedra. This structural motif enables precise control over metal–oxygen bonding, electronic conductivity, and redox behavior—key parameters governing OER activity and stability under acidic conditions [52, 53]. The Goldschmidt tolerance factor (t) is a crucial parameter influencing the distortion and durability of the perovskite lattice, defined as:

$$t=\frac{R_{A}+R_{0}}{\sqrt{2}\left(R_{B}+R_{0}\right)}$$

where R_A_, R_B_, and R_O_ represent the ionic radii of the A‐site cation, B‐site cation, and O_2_, respectively. A tolerance factor value between 0.9 and 1.0 signifies a cubic or c, stable structure; deviations from this range result in disordered structures, adversely impacting conductivity and surface activity, hence diminishing catalyst performance [26, 39]

The electronic configuration of the B‐site transition metal in perovskite oxides, particularly the occupancy of the e_g_ orbital plays a critical role in determining OER activity. Both theoretical and experimental studies suggest that an e_g_ occupancy close to unity (e.g., e_g_ ≈ 1) is often optimal, as it correlates with favorable adsorption energies for key oxygen intermediates (OH*, O*, and OOH*), thereby enhancing OER kinetics. This principle guides the rational design and doping strategies of B‐site cations to improve catalytic performance, especially in acidic environments [33, 54, 55].

While simple ABO_3_ perovskites provide a foundational platform, structurally derived variants offer additional degrees of freedom. Layered Ruddlesden–Popper (RP) phases, with the general formula A_n+1_B_n_O_3n+1_, have attracted increasing attention due to their anisotropic ion transport, enhanced surface accessibility, and ability to accommodate lattice strain. Representative iridium‐based RP compounds such as Sr_2_IrO_4_ (n = 1) and Sr_4_Ir_3_O_10_ (n = 3) exhibit high activity and durability in acidic OER, aided by weak interlayer bonding that facilitates surface reconstruction and cation redistribution during operation. These dynamic structural features are particularly advantageous under PEM‐relevant conditions, where adaptive surface evolution often governs long‐term performance [56, 57, 58].

Double perovskites (A_2_BB'O_6_) and their distorted variants offer additional opportunities for tuning catalytic properties. The coexistence of two different B‐site cations enables synergistic effects and improved charge transfer. However, lattice distortions can induce strain that modifies the electronic structure at active sites, potentially boosting both catalytic activity and corrosion resistance [59, 60, 61].

The oxygen evolution reaction (OER) on perovskite oxides generally proceeds through the adsorbate evolution mechanism (AEM) or the lattice oxygen mechanism (LOM), while the recently proposed oxide pathway mechanism (OPM) has emerged as an alternative route. In the case of a perovskite, the dominant reaction pathway is strongly influenced by the electronic structure, particularly the metal–oxygen covalency, oxygen‐vacancy concentration, and the relative position of the O 2p band center [62].

In the conventional AEM mechanism, OER proceeds through sequential proton‐coupled electron transfer involving the adsorbed intermediates *OH and *OOH. Since the reaction occurs exclusively on surface metal sites without participation of lattice oxygen, AEM generally provides good structural stability. However, its activity is fundamentally limited by the linear scaling relationship between the adsorption energies of the intermediates, resulting in a theoretical overpotential limit of approximately 370 mV [36, 62, 63]. In contrast, the LOM involves the direct participation of lattice oxygen in O–O bond formation. This mechanism bypasses the formation of *OOH intermediates by involving lattice oxygen in O_2_ evolution, thereby generating oxygen vacancies that can compromise the structural stability of the crystal lattice [36, 64]. Perovskite oxides are particularly attractive because their flexible crystal structure enables precise tuning of these mechanisms through compositional engineering. For example, increasing Sr incorporation in layered Ruddlesden–Popper perovskites promotes oxygen ligand‐hole formation, enhances Co–O covalency, and shifts the OER pathway from AEM toward LOM, resulting in significantly improved intrinsic activity. Likewise, oxygen‐defect engineering in La_x_Sr_1__−__x_CoO_3__−_δ enables continuous tuning between AEM and LOM while balancing catalytic activity and stability [65]. However, continuous participation of lattice oxygen may generate oxygen vacancies, induce extensive surface reconstruction, and eventually promote structural degradation and cation dissolution, making long‐term stability a major challenge [63, 65]. The recently proposed oxide pathway mechanism (OPM) facilitates direct O–O coupling between adjacent adsorbed oxygen species, circumventing the OOH scaling limitation and enabling O_2_ evolution without disrupting the thermodynamically stable crystal lattice [36, 66]. Representative examples include CaCu_3_Fe_4_O_12_, where incorporation of Fe^4^^+^ and Cu^2^^+^ shortened the Fe–O–Fe bond distance and enabled direct O–O coupling, as well as quadruple perovskite LaMn_7_O_12_, in which the neighboring Mn active sites promoted the OPM pathway [67, 68]. However, these studies were performed in alkaline electrolytes (1 M KOH and 0.1 M KOH, respectively).

The perovskite oxides generally undergo significant surface reconstruction during OER, making a detailed understanding of their dynamic surface evolution essential for designing stable OPM‐based catalysts [7]. Choosing a right doping element could be the key to create OPM‐favoring catalysts. Extending this concept to acid‐stable perovskite electrocatalysts therefore remains a promising but largely unexplored research direction.

### 3.2 Design Principles

The activity of perovskite electrocatalysts is commonly interpreted using the Sabatier principle, in which the adsorption strength of oxygenated intermediates governs the OER kinetics [69]. However, besides the conventional adsorbate evolution mechanism, lattice oxygen participation has been identified for many oxide catalysts, highlighting the importance of both surface adsorption properties and bulk electronic structure in catalyst design. Strategic A‐ and B‐site substitutions are vital for optimizing OER performance in perovskite structures. While A‐site doping influences lattice parameters, oxygen vacancy concentration, and phase stability, B‐site modification directly alters the nature of catalytic centers.

Based on the current understanding of acidic OER, several key design principles have emerged for the development of high‐performance perovskite electrocatalysts. First, the electronic structure of the B‐site should be optimized to achieve a favorable adsorption strength of OER intermediates while maintaining high electrical conductivity, with descriptors such as orbital occupancy and metal–oxygen covalency serving as important guidelines [70] Second, the catalyst must exhibit sufficient structural stability under strongly acidic (pH ≈ 3) and oxidizing conditions by minimizing cation dissolution while preserving the integrity of the perovskite framework [71]. Third, controlled surface reconstruction should be promoted to generate highly active surface species without excessive degradation of the bulk material, thereby balancing intrinsic activity and durability [72]. Finally, efficient electronic coupling between the bulk perovskite and the reconstructed surface is essential to maintain charge transport and sustain catalytic performance during long‐term operation [35, 37]. Together, these principles provide a framework for the rational design of next‐generation perovskite‐based electrocatalysts for acidic oxygen evolution.

Nevertheless, a key challenge in acidic OER is balancing catalytic activity and long‐term stability. Increasing the intrinsic activity of perovskites by enhancing metal–oxygen covalency or facilitating surface reconstruction often accelerates cation dissolution and structural degradation under acidic conditions [70]. Conversely, highly stable oxides generally exhibit lower catalytic activity due to weaker surface reactivity [69]. Consequently, current design strategies aim to identify an optimal compromise by promoting controlled surface reconstruction, in which for example, a highly active surface layer is formed while the underlying perovskite framework remains sufficiently stable to maintain the required catalytic performance [72].

Excessive oxidation of the active metal–oxygen framework can further intensify catalyst degradation. For Ir‐ and Ru‐containing perovskites, the literature covers strategies include regulating the oxidation state of the active metal centers through compositional engineering, suppressing lattice‐oxygen oxidation, stabilizing strongly connected BO_6_ frameworks, and controlling surface reconstruction to generate active surface species while preserving the structural integrity of the bulk catalyst [26, 42]. For example, the incorporation of a small amount of Pt into monoclinic SrIrO_3_ was reported to reduce the oxidation state of Ir and partially suppress excessive Ir oxidation, thereby alleviating Ir dissolution and improving catalyst stability during acidic OER [73]. Similarly, partial replacement of lattice oxygen with sulfur in monoclinic SrIrO_3_ generated favorable oxygen vacancies and reduced the oxidation state of Ir, resulting in a lower OER overpotential and stable operation for more than 20 h at 10 mA cm^−^^2^ in 0.5 M H_2_SO_4_ [74]. In contrast, degradation of non‐PGM perovskites under acidic OER conditions is generally dominated by rapid A‐ and B‐site cation leaching, lattice destabilization, and framework collapse rather than by the formation of stable over‐oxidized active species. Consequently, only Ir‐ and Ru‐based perovskites have so far demonstrated sufficient stability for sustained operation in acidic media [28, 42]. For non PGM structure, Fe incorporation into cobalt‐based perovskites enhances charge stability, promotes oxygen‐defect formation, and stabilizes B‐site Co in a lower oxidation state. These effects suppress the formation of inactive amorphous oxide phases and extend the thermodynamic metastable potential window, ultimately improving the structural stability of the perovskite during OER [75]. Another effective strategy for non‐PGM perovskites is A‐site cation deficiency engineering. Appropriate A‐site deficiency has been shown to regulate the oxidation state of B‐site transition metals while promoting the formation of oxygen vacancies without disrupting the perovskite framework. For example, La_0_._95_FeO_3_ exhibited enhanced OER activity due to a moderate increase in surface oxygen vacancies and Fe^4^^+^ species [76]. Similarly, A‐site double‐cation‐deficient (La_0_._6_Sr_0_._4_)_1__−__x_Co_0_._8_Fe_0_._2_O_3__−_δ and (Ba_0_._5_Sr_0_._5_)_1__−__x_Co_0_._8_Fe_0_._2_O_3__−_δ generated a higher concentration of oxygen vacancies and higher oxidation states of the B‐site cations than single A‐site‐deficient materials [77]. However, the degree of A‐site deficiency must be carefully optimized, as excessive cation deficiency can destabilize the perovskite structure and promote impurity‐phase formation [78].

From a scale‐up perspective, catalyst design should not only maximize intrinsic activity, but also ensure compatibility with scalable synthesis routes, reproducible composition and morphology, mechanical and chemical robustness, and integration into catalyst‐coated electrodes. These structure–property relationships form the basis for  the catalyst systems discussed in the following section [5, 35, 79, 80].

Although numerous perovskite compositions have been investigated for acidic OER, only a limited number exhibit both high activity and even less long‐term stability. This section therefore addresses two key questions: Which structural characteristics distinguish the most successful acidic perovskite catalysts, and what design principles emerge from the different perovskite families to guide the development of future electrocatalysts? To address these questions, this section compares the most representative perovskite systems reported for acidic OER, highlighting the influence of crystal structure, elemental composition, electronic properties, or surface evolution on catalytic performance.

### 3.3 Strontium Iridates and Related Ruddlesden–Popper

Strontium iridates and their layered RP analogs are promising perovskite oxide catalysts for acidic OER due to their excellent corrosion resistance, intrinsic electronic conductivity, and strong lattice stability [26, 81, 82]. Despite substantial progress, the influence of bulk crystal structure on intrinsic acidic OER activity in layered perovskites remains incompletely understood. A systematic investigation of the RP series Sr_n__+__1_Ir_n_O_3__n__+__1_ (n = 1, 2, ∞) revealed a pronounced structure–activity relationship, with Sr_3_Ir_2_O_7_ (n = 2) exhibiting the most favorable performance. This compound delivered low overpotentials of 259 mV in acidic media and 296 mV in alkaline electrolyte at 10 mA cm^−^^2^ (Figure 2a), accompanied by moderate Tafel slopes of 50 and 53 mV dec^−^^1^, respectively.

**FIGURE 2:**
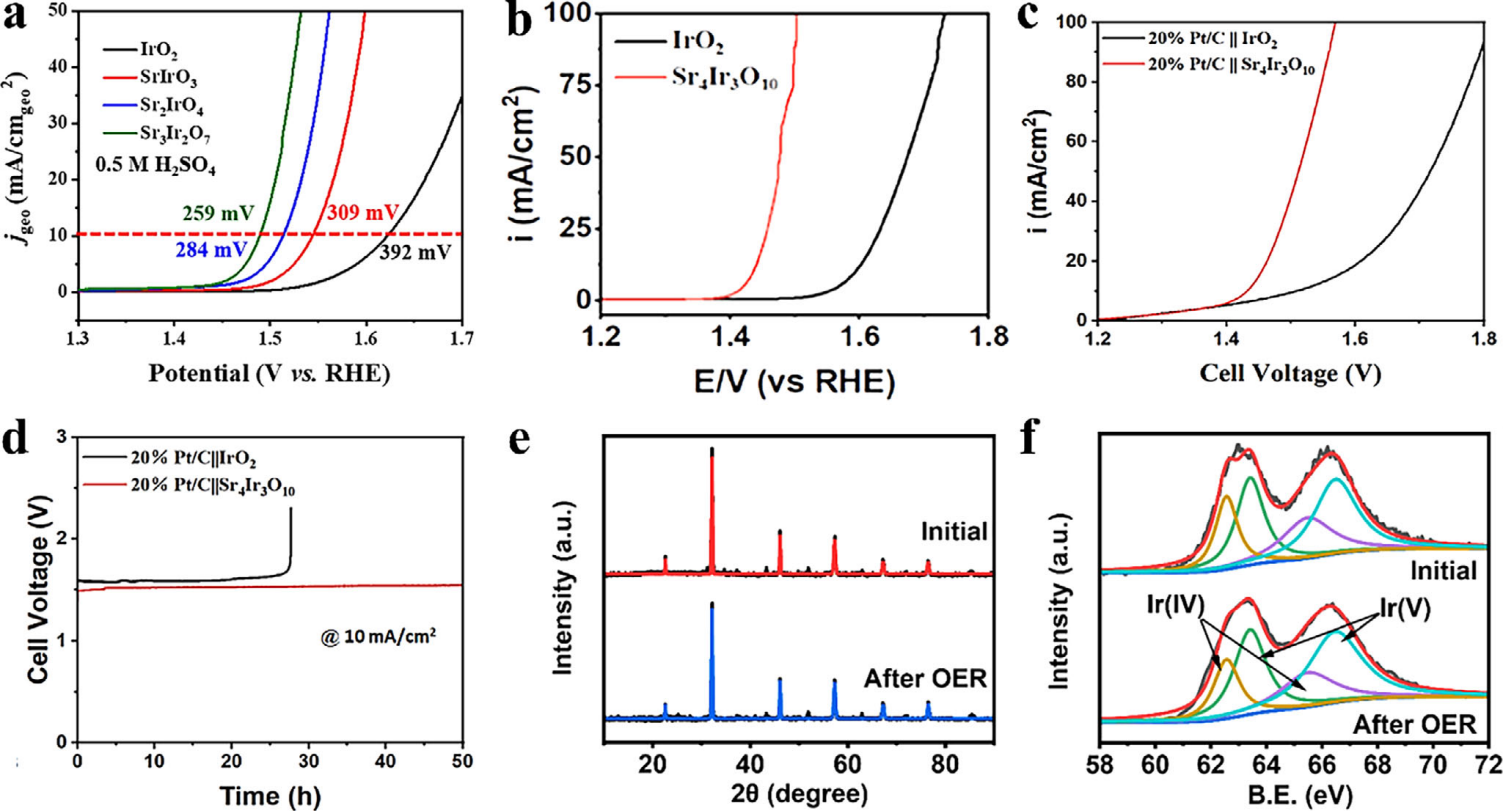
(a) OER curves for different catalysts (deposited on glassy carbon working electrode measured in 0.5 M H_2_SO_4_ using linear sweep voltammetry (LSV) from 1.2 to 1.8 V versus RHE at scanning rate of 5 mV s^−1^). Reproduced with permission [57]. Copyright 2024, Elsevier. (b) OER curves for different catalysts (deposited on glassy carbon working electrode measured in 0.1 M HClO_4_ using LSV from 1.0 to 1.8 V versus RHE at a scanning rate of 10 mV s^−1^), (c) cell voltage‐current curves of assembled PEM cell (catalyst as the anode and Pt/C with 20 wt.% Pt as the cathode), (d) corresponding galvanostatic stability curves at 10 mA cm^−2^ of assembled PEM cell (catalyst as the anode and Pt/C with 20 wt.% Pt as the cathode), (e) X‐ray diffraction (XRD) pattern and (f) XPS spectra of Sr_4_Ir_3_O_10_ before and after galvanostatic stability testing. Reproduced with permission [56]. Copyright 2024, Elsevier.

The superior activity of Sr_3_Ir_2_O_7_ originates from a combination of optimized electronic structure and layered architecture. Density functional theory calculations indicate that tuning of the Ir d‐band center weakens the binding strength of oxygen intermediates, thereby lowering the OER overpotential. Concurrently, the intermediate number of perovskite layers in the n = 2 RP phase provides enhanced electronic conductivity relative to n = 1, while avoiding the structural rigidity of three‐dimensional SrIrO_3_. In addition, SrO‐terminated surfaces modulate the local electronic environment of surface Ir sites, accelerating reaction kinetics and improving intrinsic activity. These features collectively highlight how layered RP structures enable fine control over electronic and structural parameters governing acidic OER [57]. While the lattice oxygen mechanism (LOM) offers low energy barriers for OER, it can introduce positively charged vacancies, destabilizing the structure. The introduction of RP perovskite (Sr_4_Ir_3_O_10_), characterized by dynamic interlayer oxygen migration, may address this issue by providing elevated activity via LOM while maintaining significant structural stability. Furthermore, this Sr_4_Ir_3_O_10_ has been used in proton exchange membrane water electrolysis (PEMWE) for the first time. This distinctive structure employs a stepwise O_2_ conversion from adsorbed water to interstitial oxygen and eventually to lattice oxygen, providing excellent activity and structural stability in acidic environments, hence assuring robust catalysts for PEMWE applications. The synthesized Ruddlesden‐Popper perovskite catalyst required a potential of 1.435 V to achieve a current density of 10 mA cm^−2^ (Figure 2b), demonstrating a stability of 35 h and a Tafel slope of 66.1 mV/dec. The Sr_4_Ir_3_O_10_‐Pt/C based PEMWE demonstrates a voltage of 1.47 V at 50 mA cm^−2^ (Figure 2c), exhibiting 50 h of stability without significant voltage degradation (Figure 2d); moreover, the structure and morphology of Sr_4_Ir_3_O_10_ remain unchanged after 50 h of continuous operation (Figure 2e,f). The following factors contribute to the stability of this catalyst: Low Fermi level leads to a decreased energy barrier, hence improving OER kinetics, (ii) Dynamic short‐range O_2_ migration, attributable to the layered structure, facilitates rapid and efficient O_2_ evolution, (iii) Elevated intrinsic activity from active lattice O_2_, (iv) Refined reaction pathway, Synergistic effects, (v) Continuous replenishment of lattice oxygen prevents structural collapse, (vi) The self‐healing process preserves the SrO layer integrity, and (vii) Optimal synthesis settings that augment overall efficiency [56].

Beyond conventional RP phases, alternative layered architectures have also demonstrated promising acidic OER performance. A honeycomb‐layered strontium iridate, SrIr_2_O_6_, was developed to combine high intrinsic activity with structural stability [83]. Unlike many RP iridates, SrIr_2_O_6_ predominantly follows the adsorbate evolution mechanism (AEM), thereby avoiding the formation of oxygen vacancies that can accelerate degradation. The catalyst exhibited an overpotential of 303 mV at 10 mA cm^−^^2^, a low Tafel slope of 44.2 mV dec^−^^1^ (Figure 3a), and remarkable durability exceeding 300 h (Figure 3b). Its honeycomb layered structure (Figure 3c) preserves crystallinity during operation, while ultrashort Sr–O bonds suppress Sr^2^^+^ leaching and enhance lattice stability. Highly active edge‐sharing IrO_6_ motifs further promote favorable Ir–O interactions and rapid OER kinetics, resulting in sustained performance without amorphization.

**FIGURE 3:**
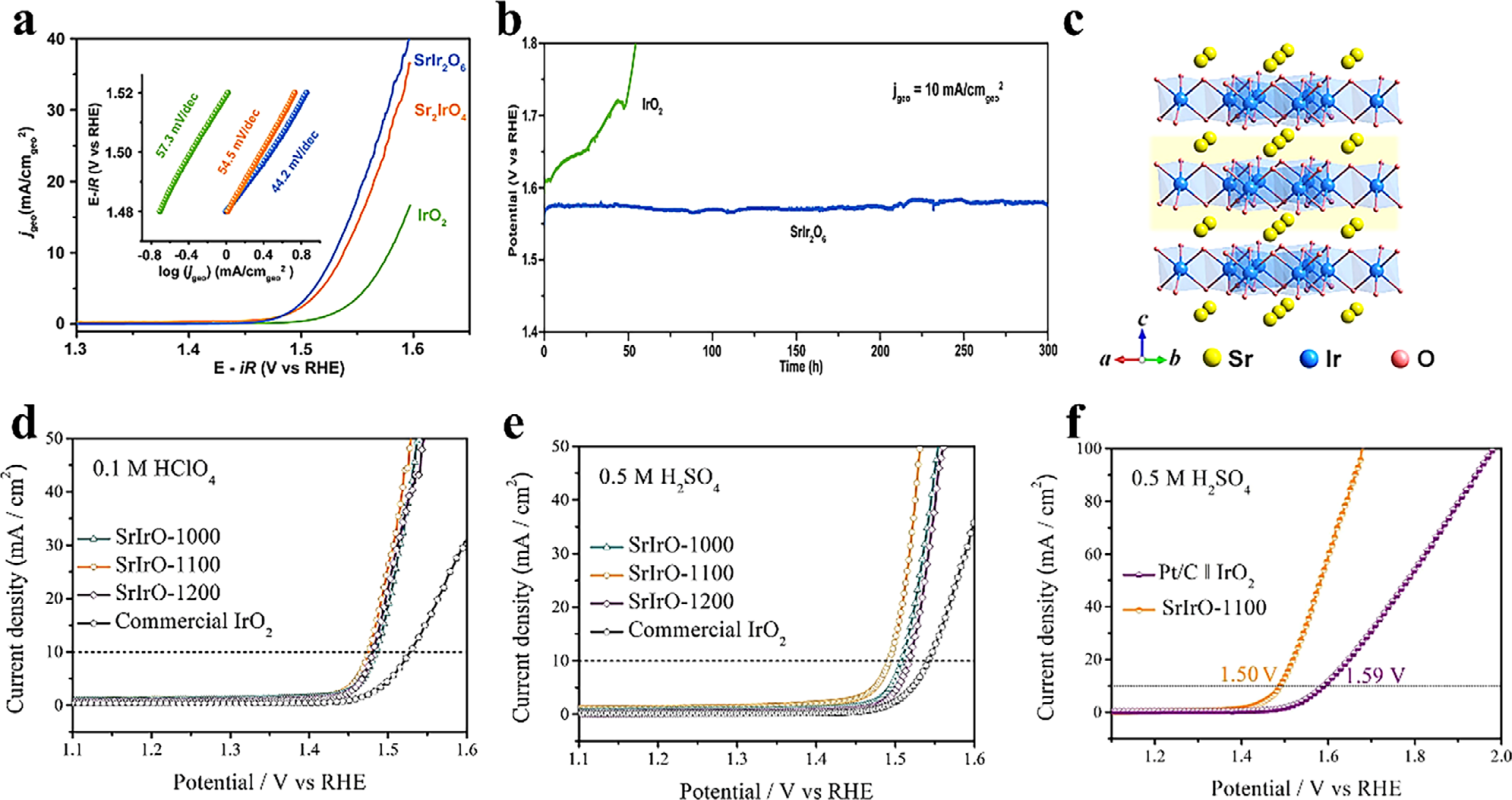
(a) OER curves from different catalysts (deposited on glassy carbon working electrode measured in 0.1 M HClO_4_ using LSV from 0.8 to 1.65 V versus RHE at scanning rate of 1 mV s^−1^) with corresponding Tafel slopes inserted in the figure, (b) Chronopotentiometric stability curves of different catalysts (deposited on 0.3 × 0.3cm^2^ carbon paper at 10 mA cm^−2^ current density without iR‐drop compensation versus RHE) and (c) honeycomb layered SrIr2O6 crystal structure. Reproduced with permission [83]. Copyright 2023, John Wiley and Sons. (d) OER curves from different catalysts (deposited on glassy carbon working electrode measured in 0.1 M HClO_4_ using LSV from 1.0 to 1.6 V versus RHE scan rate ‐), e) OER curves from different catalysts (deposited on glassy carbon working electrode measured in 0.5 M H_2_SO_4_ using LSV from 1.0 to 1.6 V versus RHE scan rate ‐), and (f) OER polarization curve for different catalysts (deposited on 1 × 1 cm^2^ Ti‐meshes catalyst loading 1 mg cm^−2^ used for both anode and cathode, measured in 0.5 M H_2_SO_4_ using LSV from 1.0 to 2.0 V versus RHE scan rate ‐). Reproduced with permission [84]. Copyright 2023, American Chemical Society.

Hybrid perovskite architectures have also been explored to overcome the intrinsic instability of conventional three‐dimensional iridates. A composite catalyst comprising quasi‐two‐dimensional Sr_2_IrO_4_ and three‐dimensional 6H‐SrIrO_3_ (denoted SrIrO‐1100) was designed to exploit complementary structural features [84]. In this system, face‐shared IrO_6_ octahedra in 6H‐SrIrO_3_ provide highly active surface sites, while the layered Sr_2_IrO_4_ alloy enables controlled Sr^2^^+^ leaching without collapse of the IrO_6_ framework. This hybrid structure enhances electron transport, increases active site exposure, and lowers the kinetic energy barrier for OER. The SrIrO‐1100 catalyst achieved overpotentials of 245 mV and 263 mV at 10 mA cm^−^^2^ in HClO_4_ and H_2_SO_4_ electrolytes, respectively (Figure 3d,e), with stable operation over 40 h. In a two‐electrode configuration, the catalyst delivered 10 mA cm^−^^2^ at 1.50 V (Figure 3f) and maintained stable performance for 10 h, demonstrating the potential of hybrid perovskite architectures for acidic OER under realistic operating conditions.

### 3.4 Lanthanum Strontium Manganite

La_1__−__x_Sr_x_MnO_3_ (LSM) is a perovskite oxide in which La and Sr occupy the A‐site and Mn resides at the B‐site. Owing to its high electronic conductivity and excellent thermal stability, LSM has been widely investigated as an electrode material for energy conversion and electrochemical applications^59, 60^. Despite these advantages, LSM exhibits limited stability under aggressive operating conditions, primarily due to strontium surface segregation and the formation of oxygen vacancies, both of which progressively degrade long‐term performance.

To mitigate these degradation pathways, various surface modification strategies have been explored, including the incorporation of heteroatoms such as TiO_2_ and other stabilizing elements. These approaches have been shown to suppress Sr segregation, regulate defect chemistry, and enhance electrochemical durability under demanding conditions [38, 85, 86]. As a result, LSM‐based perovskites have been extensively employed as electrocatalysts for the oxygen evolution reaction in alkaline media, where they exhibit favorable activity and stability [87, 88]. Beyond aqueous electrochemistry, LSM is a well‐established cathode material in solid oxide fuel cells, benefiting from its compatibility with commonly used electrolytes, high electronic conductivity, and thermal robustness at elevated temperatures [89, 90]. In contrast, despite its widespread use in alkaline OER and solid oxide electrochemical devices, LSM has not, to date, been reported as an effective catalyst for acidic OER. This absence is most likely attributable to insufficient chemical stability under strongly acidic conditions, which remains a critical barrier for its application in proton exchange membrane water electrolysis.

### 3.5 Doped Perovskites

Doped perovskites are a broad class of ABO_3_‐type materials in which the A‐site, B‐site, and/or oxygen sublattice is partially substituted to tailor electronic structure, defect chemistry, and catalytic performance. A‐site doping primarily influences lattice parameters and structural stability, while B‐site substitution introduces new redox couples that modulate electronic configuration and promote oxygen vacancy formation. Oxygen‐site doping further affects metal–oxygen bond strength, lattice basicity, and charge distribution. Collectively, these strategies provide powerful means to optimize activity–stability trade‐offs in oxygen evolution catalysis [28, 91, 92].

Ru‐ and Ir‐based alloys are among the most active perovskite OER catalysts; however, achieving simultaneous high activity and durability under acidic conditions remains challenging. One effective strategy involves A‐site alkali‐metal doping to stabilize the perovskite framework and tune electronic structure. For example, Na‐doped ruthenate perovskites, Sr_0_._95_Na_0_._05_RuO_3_ and Sr_0_._90_Na_0_._10_RuO_3_, exhibited exceptionally low overpotentials of 120 mV at 0.5 mA cm^−^^2^ in 0.1 M HClO_4_ (Figure 4a), along with sustained stability (Figure 4b) [93]. These enhancements arise from Na‐induced stabilization of the perovskite lattice, which lowers surface energy, increases dissolution potentials, and reduces distortion of RuO_6_ octahedra under acidic bias. Concurrently, Na doping shifts the Ru d‐band and O p‐band centers toward the Fermi level, increasing the effective oxidation state of Ru (Figure 4c), weakening Ru–adsorbate interactions, and suppressing cation leaching. As a result, catalytically versatile and durable active sites are generated under operating conditions.

**FIGURE 4:**
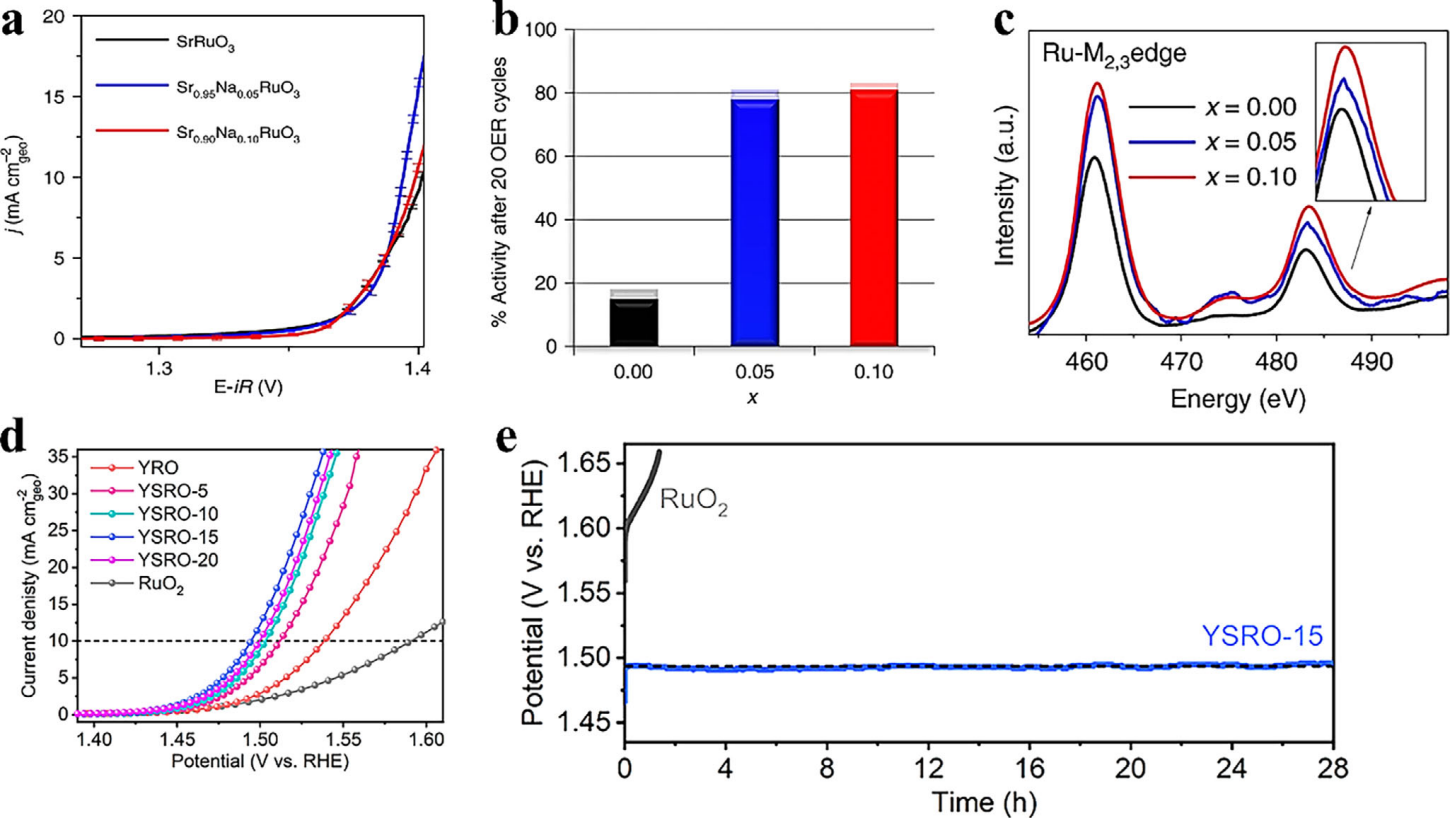
(a) OER curves from different catalysts (deposited on glassy carbon electrode measured at 1600 rpm rotation with rotating disc electrode (RDE) in O_2_‐saturated 0.1 M HClO_4_ using cyclic voltammetry (CV) from 1.1 to 1.5 V or 1.1 to 1.7 V versus RHE at scanning rates of 10 mV s^−1^ or 1 mV s^−1^), (b) corresponding bar chart of percentage activity after 20 OER cycles including relative standard deviation as shadow bar and (c) Ru M_2,3_‐edge XANES spectra for Sr_0.90_Na_0.10_RuO_3_ (red), SrRuO_3_ (black), and Sr_0.95_Na_0.05_RuO_3_ (blue). The zoom‐in shows ∼461 eV region featuring an upward displacement of the SrRuO_3_ spectrum to improve clarity. Reproduced with permission [93]. Copyright 2023, John Wiley and Sons. (d) OER curves from different catalysts (deposited on glassy carbon electrode measured in O_2_‐saturated 0.5 M H_2_SO_4_ using CV from 1.3 to 1.6 V versus RHE at scanning rate of 5 mV s^−1^), and (e) chronopotentiometric stability curves of RuO_2_ and YSRO‐15 (measured at 10 mA cm^−2^ versus RHE). Reproduced with permission [94]. 2019, Springer Nature.

Y_2_Ru_2_O_7_ has good OER performance in acidic electrolytes; yet, its electrons are confined owing to internal pressures, resulting in insulating behavior that hinders efficient charge transfer between reacting molecules and the electrolyte, rendering it unfavorable for OER. This issue is addressed by altering the electrical structure by partial A‐site substitution. The partial substitution of Y^3+^ with Sr^2+^ alters the electronic structure, enhancing its suitability for OER. The novel material, Y_1.7_Sr_0.3_Ru_2_O_7_ (YSRO‐15), exhibits superior OER activity, characterized by an overpotential of 264 mV at a current density of 10 mA cm^−2^ in a 0.5 M H_2_SO_4_ electrolyte (Figure 4d), with a stability duration of 28 h (Figure 4e). The following factors contribute to the catalyst's performance: The replacement of Y^3+^ with Sr^2+^ induces charge compensation that influences the oxidation state of Ru, increases Ru‐O covalency, and stabilizes reaction intermediates, (ii) Enhanced O_2_ adsorption energies from Sr doping adjust electronic correlations and d‐orbital occupancy at the Ru active site, resulting in optimized Gibbs free energy differentials and reduced OER overpotential, (iii) Increased surface O_2_ vacancies and the existence of reactive O_2_ species, such as O^−^ and O_2_^−^, serve as active sites for OER as a result of strontium doping, (iv) Robust metal‐oxygen bonding yields significant stability with little degradation, and (v) Reduced charge transfer resistance enhances electron transfer efficiency [94].

Although ruthenium‐based catalysts generally outperform iridium‐based analogues in terms of activity, their stability under acidic conditions remains a critical concern. In this context, partial substitution at the A‐site of Y_2_Ru_2_O_7_ with Co provides an effective route to balance activity and durability. The Y_1_._75_Co_0_._25_Ru_2_O_7_−δ (YCR) electrocatalyst exhibited an overpotential of 275 mV at 10 mA cm^−^^2^ in 0.5 M H_2_SO_4_ (Figure 5a) and retained stable performance over 2000 cycles (Figure 5b). ^72^ The following factors contribute to the efficacy of this catalyst: Modulated Ru electronic structure resulting from Co doping induces charge redistribution that impacts Ru^4+^ electronic configuration, oxidation state, enhances Ru‐O covalency, and improves metal‐oxygen orbital hybridization, hence enabling OER, (ii) Enhanced adsorption energies from Co doping lead to d‐orbital occupancy and electrical interactions at the Ru active site, optimizing the Gibbs free energy variations among O_2_ intermediates and reducing OER overpotential, (iii) Reduced charge transfer resistance leads to improved kinetics (Figure 5c), (iv) Augmented active oxygen species, including O^−^ and O_2_^−^, together with surface O_2_ vacancies resulting from Co doping, and (v) Negligible degradation owing to robust metal‐O_2_ interactions [95].

**FIGURE 5:**
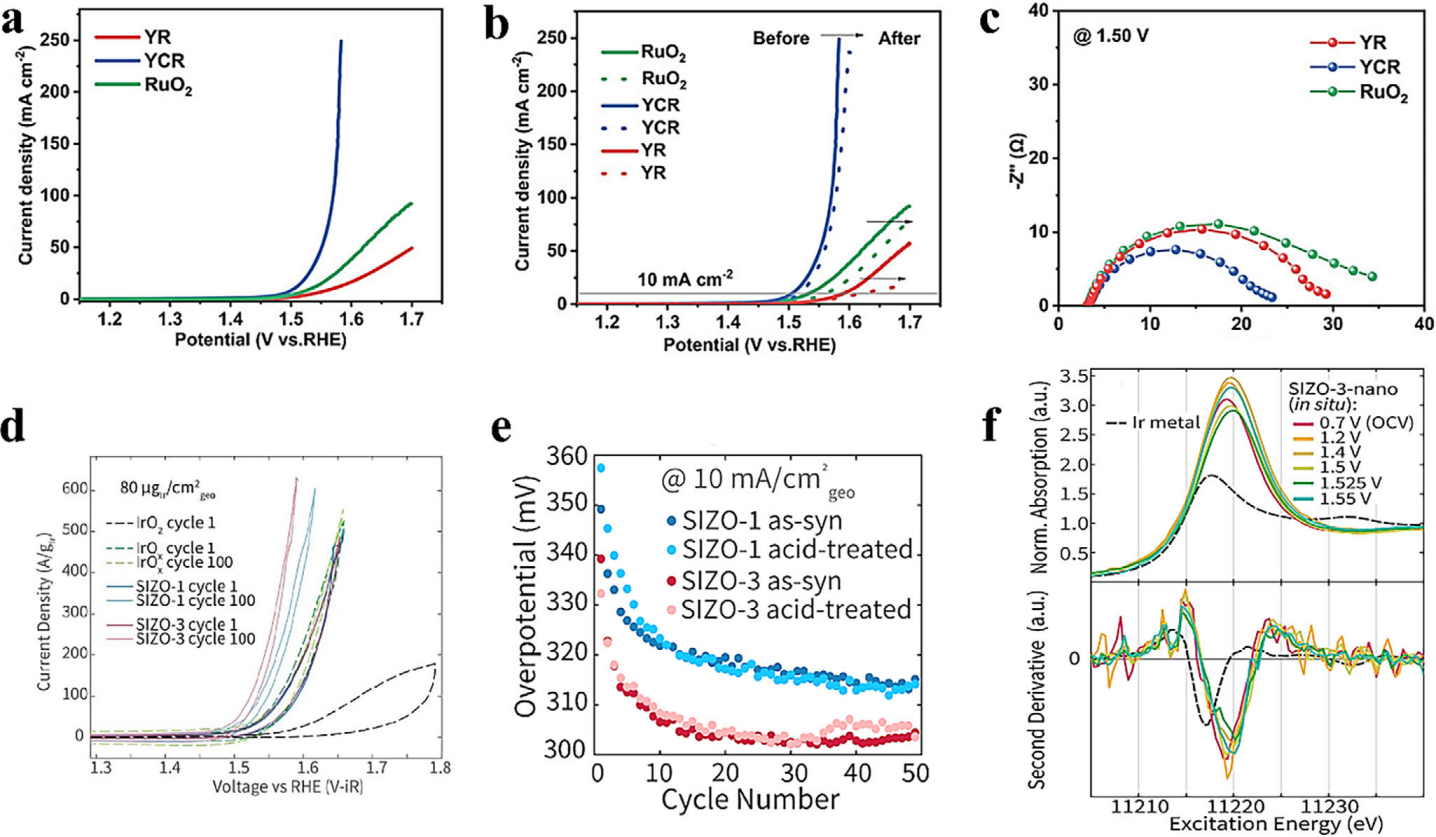
(a) OER curves from different catalysts (deposited on polished RDE working electrode measured at 1600 rpm rotation with RDE in O_2_‐saturated 0.5 M HClO_4_ using LSV from 1.1 to 1.7 V versus RHE at scanning rate of 5 mV s^−1^), (b) corresponding OER curves initial (bold) and after 200 cycles (dashed), and (c) electrochemical impedance spectroscopy (EIS) Nyquist plots of different catalysts at 1.5 V (versus RHE) OER potential. Reproduced with permission [95]. Copyright 2021, American Chemical Society. (d) CV curves (in 0.1 M HCLO_4_ at 20 mV s^−1^ scan rate versus RHE) of different catalysts with catalyst ink loading of 80 µg Ir cm^−2^_geo_ (rutile IrO_2_ not stable over 100 cycle range), (e) overpotential evolution over the first 50 cycles to reach 10 mA cm^−2^ current density (0.1 M HCLO_4_), and (f) in situ Ir L_III_‐edge X‐ray absorption spectroscopy (XAS) spectra SIZO‐3 catalyst reveal normalized absorption and its second‐derivative in the XANES region. Reproduced with permission [96]. Copyright 2023, John Wiley and Sons.

B‐site doping has also proven effective for improving both performance and sustainability in iridium‐based perovskites. Orthorhombic SrIrO_3_ is a benchmark acidic OER catalyst due to its high activity and stability; however, its synthesis is energy intensive and its mechanistic behavior remains complex. Doping the B‐site with zinc not only decreases the synthesis energy but also lowers costs, since zinc is plentiful and has a low global warming potential. Substitution of Ir with Zn addresses these challenges by reducing synthesis energy and material cost, given the abundance and low environmental impact of Zn. The resulting polycrystalline SrIr_0_._8_Zn_0_._2_O_3_ (SIZO) electrocatalyst exhibits progressively enhanced OER activity upon electrochemical cycling (Figure 5d) together with excellent stability [96]. Operando analyses reveal that cycling induces surface reconstruction and amorphization of the near‐surface region (Figure 5e), accompanied by an increase in the Ir oxidation state (Figure 5f). Zn substitution further stabilizes the lattice, facilitates scalable synthesis, and alters the local coordination environment of Ir, thereby tuning the d‐band center and promoting favorable O–O bond formation pathways during OER [96].

### 3.6 Unconventional Ir‐Based Perovskites

In addition to conventional Sr–Ir‐based perovskites, unconventional iridium‐based perovskite architectures in which Sr is replaced by alternative cations have attracted increasing attention for acidic water electrolysis owing to their distinct crystal structures and electronic configurations [37, 97]. Given the high cost and limited availability of iridium, catalysts exhibiting high Ir mass activity are essential for achieving practical performance while minimizing noble‐metal loading. Although extensive efforts have focused on optimizing Sr–Ir perovskites through diverse synthesis routes, the mechanistic origins of their activity and durability have often remained unclear.

A notable example is the IrO_x_/9R‐BaIrO_3_ catalyst, in which ∼1 nm IrO_x_ nanoparticles are uniformly anchored onto a hexagonal 9R‐BaIrO_3_ perovskite support. This architecture delivers a high Ir mass activity of 168 A gIr^−^^1^, together with a low overpotential of 230 mV at 10 mA cm^−^^2^ in 0.5 M H_2_SO_4_ (Figure 6a), while maintaining excellent operational stability (Figure 6b) [98]. During OER the IrO_x_ nanoparticles undergo progressive transformation from Ir^4^^+^‐rich amorphous IrO_x_Hy species to Ir^5^^+^‐dominated IrO_x_ layers composed of IrO_6_ octahedra. These highly oxidized surface motifs generate electrophilic oxygen species that facilitate water activation and oxygen–oxygen bond formation. Concurrently, an increased density of states near the Fermi level and reduced charge‐transfer resistance (Figure 6c) enable rapid electron transport. Despite substantial Ba leaching under acidic conditions, the IrO_6_ framework and the core 9R‐BaIrO_3_ structure remain largely intact (Figure 6d), resulting in minimal overpotential change even after 48 h of operation (Figure 6e). The formation of stable amorphous IrO_x_ surface layers supported by a robust crystalline backbone thus reconciles high activity with long‐term durability.

**FIGURE 6:**
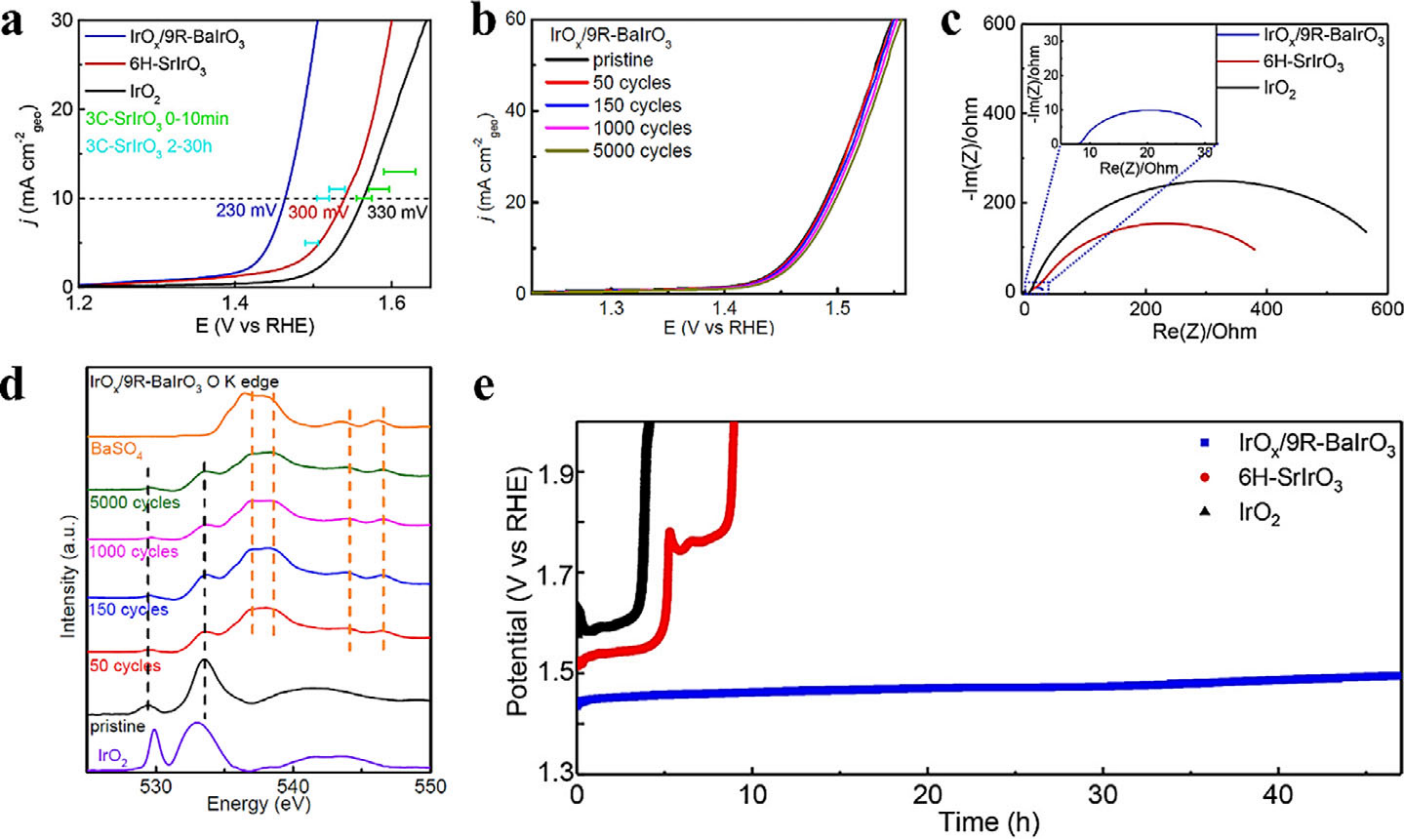
(a) OER curves from different catalysts (deposited on 3 mm glassy carbon working electrode measured in O_2_‐saturated 0.5 M H_2_SO_4_ using LSV from 1.2 to 1.7 V versus RHE at scan rate of 5 mV s^−1^; data iR corrected), (b) CV polarization curves of IrO_x_/9R‐BaIrO_3_ at 10 mA cm^−2^ current density (measured in O_2_‐saturated 0.5 M H_2_SO_4_), the overpotential is ∼10 mV increased from pristine to 5000 cycle curve, (c) EIS Nyquist plot of different catalysts at 1.53 V (versus RHE), (d) O K‐edge XANES spectra obtained from IrO_x_/9R‐BaIrO_3_, IrO_2_ and BaSO_4_ after various cycles. Minor BaSO_4_ was formed and absorbed on 9R‐BaIrO_3_ attributed to Ba leaching during acidic OER, and (e) Chronopotentiometric stability curve of different catalysts at 10 mA cm^−2^ (measured in 0.5 M H_2_SO_4_, measurement conducted using carbon paper catalysts support). Reproduced with permission [98]. Copyright 2024, American Chemical Society.

The role of A‐site leaching and surface amorphization in acidic OER has been further elucidated using Ca_2__−__x_IrO_4_ as a model system. Previous studies suggested that amorphous surface layers formed during Sr leaching enhance activity, yet the relationship between amorphization, stability, and electrolyte chemistry remained poorly understood. In Ca_2__−__x_IrO_4_, controlled surface amorphization was induced by cyclic voltammetry in 0.5 M H_2_SO_4_, producing a low overpotential of 236 mV at 10 mA cm^−^^2^ (Figure 7b) after only ten cycles (Figure 7a) [98]. In contrast, no amorphous layer formed even after 5000 cycles in 1 M HClO_4_ (Figure 7c). Remarkably, the catalyst maintained stability for over 100 h in H_2_SO_4_ and nearly 300 h in HClO_4_ (Figure 7d). These observations highlight the critical influence of electrolyte anions: strongly coordinating SO_4_^2^^−^ species promote surface amorphization and enhanced activity through improved charge transfer and increased active‐site density, but also accelerate degradation, whereas weakly coordinating ClO_4_^−^ anions preserve crystallinity and structural integrity at the expense of lower activity. X‐ray absorption fine structure analysis confirms that the IrO_6_ octahedral framework remains intact in both cases (Figure 7e), demonstrating resilience against complete lattice collapse despite significant Ca leaching. The presence of highly oxidized Ir^5^^+^ species further contributes to high intrinsic OER activity.

**FIGURE 7:**
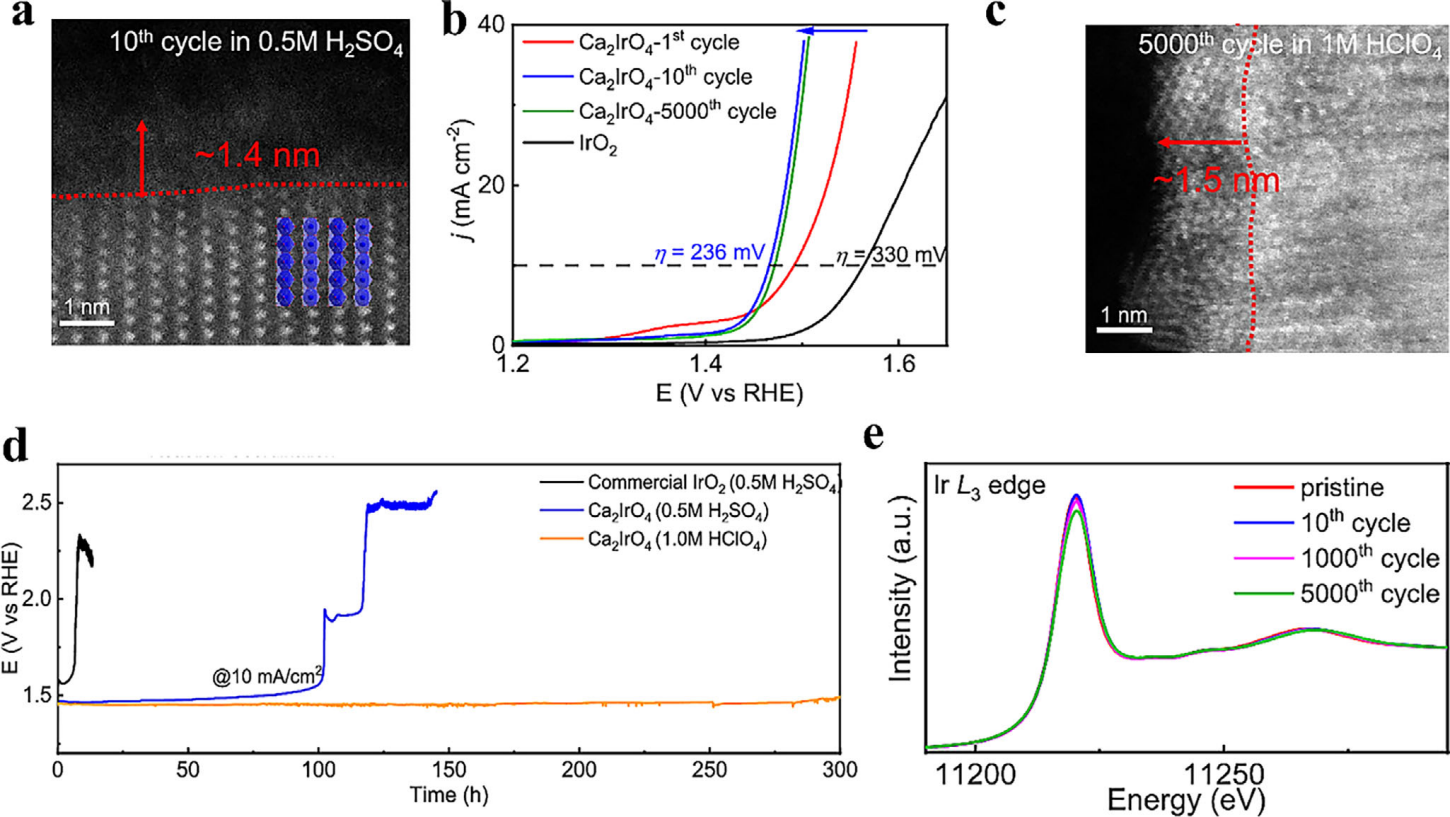
(a) High‐angle annular dark field scanning transmission microscopy (HAADF‐STEM) image of pristine Ca_2‐_ *_x_* IrO_4_ after 10 CV cycles in 0.5 M H_2_SO_4_ (blue ball: Ir, green ball: Ca, red ball: O), (b) OER curves from IrO_2_ and Ca_2_IrO_4_ after different cycles (deposited on 3 mm glassy carbon working electrode measured in 0.5 M H_2_SO_4_ using LSV from 1.2 to 1.7 V versus RHE at scan rate of 5 mV s^−1^, with iR compensation), (c) lattice‐resolved HAADF‐STEM image of pristine Ca_2‐_ *_x_* IrO_4_ after 500 CV cycles in 1 M HCLO_4_., (d) chronopotentiometric stability curve of different catalysts at 10 mA cm^−2^ (measured in 0.5 M H_2_SO_4_ and 1 M H_2_ClO_4_) and (e) Ir L_3_‐edge X‐ray absorption fine structure (XAFS) spectra of pristine Ca_2‐_ *_x_* IrO_4_ after different cycles in 0.5 M H_2_SO_4_. Reproduced with permission [98]. Copyright 2024, American Chemical Society.

To overcome the longstanding trade‐off between activity enhancement through Ir dilution and the accompanying loss of stability, mixed‐cation perovskites have been developed in which Sr is replaced by Y and Ca. The resulting Ca_2_Y_0_._2_Ir_0_._8_O_4_ catalyst achieves an overpotential of only 213 mV at 10 mA cm^−^^2^ (Figure 8a) while sustaining stable operation for more than 168 h (Figure 8b) [36]. Operando Raman spectroscopy and XAFS reveal the formation of Y‐superoxide intermediates (YOO^−^) and highly oxidized Ir^5^^+^ species during OER (Figure 8c). Y^3^^+^ substitution induces charge compensation that increases oxygen vacancy concentration and electrophilic lattice oxygen, thereby facilitating O–O bond formation. Simultaneously, robust Ir–O–Y bonding stabilizes the structure, while the Ca_2_IrO_4_ backbone provides a resilient scaffold capable of withstanding aggressive acidic conditions. Controlled formation of surface IrOx layers further accelerates OER kinetics without compromising durability.

**FIGURE 8:**
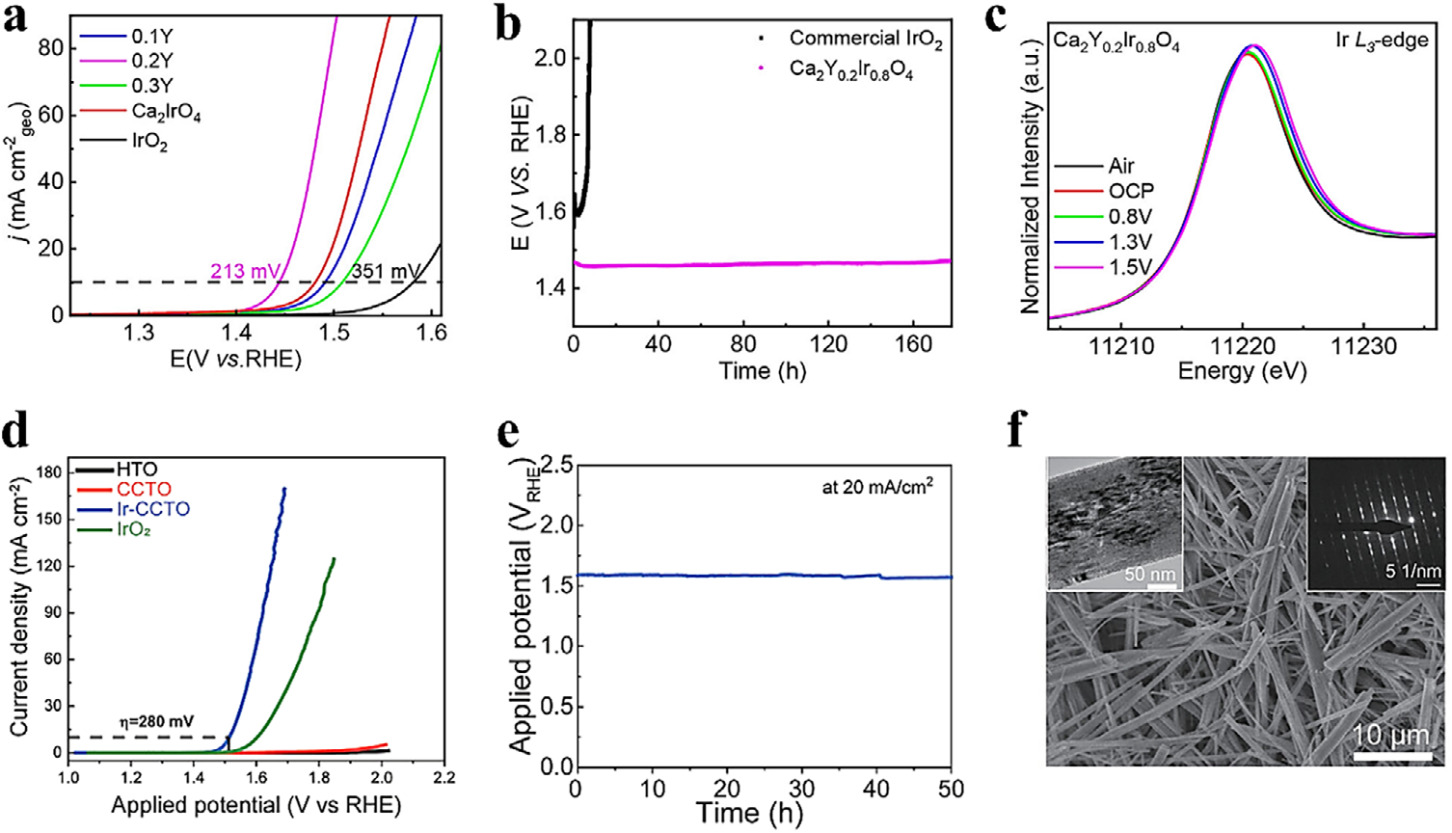
(a) OER curves from different catalysts (deposited on glassy carbon working electrode measured in 1 M HClO_4_ using LSV from 1.2 to 1.7 V versus RHE at scan rate of 5 mV s^−1^; with iR‐drop compensation), (b) chronopotentiometric stability curve of commercial IrO_2_ and Ca_2_Y_0.2_Ir_0.8_O_4_ at 10 mA cm^−2^ (measured in 1 M HClO_4_), and (c) Operando Ir L_3_‐edge XAFS spectra of Ca_2_Y_0.2_Ir_0.8_O_4_ nanocrystals at various potentials. Reproduced with permission [36]. Copyright 2022, American Chemical Society. (d) OER curves of H_2_Ti_3_O_6_ (HTO), CaCu_3_Ti_4_O_12_ (CCTO), Ir‐CCTO and IrO_2_ catalysts (deposited on 3 mm glassy carbon RDE working electrode measured in 0.1 M HClO_4_ using LSV from 1.2 to 1.65 V versus RHE at scan rate of 5 mV s^−1^), (e) chronopotentiometric stability curve of different catalysts at 20 mA cm^−2^ (measured in 0.1 H_2_ClO_4_), and (f) scanning electron microscopy (SEM) image of as‐prepared HTO nanobelts (NB), left insert shows transmission electron microscopy (TEM) image of Iridium‐CCTO NBs, right insert shows corresponding selected‐area electron diffraction (SAED). Reproduced with permission [99]. Copyright 2022, American Chemical Society.

Beyond bulk perovskite design, the choice of support material plays a decisive role in maximizing Ir utilization. Calcium copper titanate (CaCu_3_Ti_4_O_12_, CCTO), a perovskite‐type oxide with colossal permittivity (εr > 10^4^), offers high polarizability and abundant oxygen vacancies that are advantageous for charge transfer and reactant adsorption. However, pristine CCTO lacks sufficient conductivity and stability for acidic OER. These limitations can be overcome by substituting Ti^4^^+^ with Ir^4^^+^, enabled by their similar ionic radii. The resulting Ir‐CCTO catalyst exhibits a high Ir mass activity of 1000 A gIr^−^^1^ and a low overpotential of 280 mV at 10 mA cm^−^^2^ in 0.1 M HClO_4_ (Figure 8d), together with stable operation over 50 h (Figure 8e).

Ir incorporation enhances metal–oxygen covalency, reduces charge‐transfer resistance, and generates surface IrOx nanoclusters that serve as highly active OER sites.

## 4 Synthesis Strategies

The performance of acidic perovskite electrocatalysts is intrinsically linked not only to their composition and crystal structure but also to the synthesis route employed to obtain them. The choice of synthesis method determines key characteristics such as crystallinity, particle size or surface area all of which strongly influence catalytic activity. This section therefore addresses two complementary questions: Which synthesis strategies are most effective for producing high‐performance acidic perovskite electrocatalysts, and how do different preparation methods influence the structural and electronic properties that govern their catalytic performance?

### 4.1 Spray‐Flame Synthesis

While wet‐chemical synthesis routes typically yield materials governed by thermodynamic growth, gas‐phase processes enable kinetic control over particle formation, facilitating access to metastable phases, doped nanoparticles, and nanocomposites. Among these approaches, flame spray synthesis (FSS) stands out as an industrially relevant technique owing to its high throughput, cost efficiency, and scalability.

In FSS, metal precursors—commonly metal salts dissolved in alcohol–water mixtures—are atomized into a flame, where rapid solvent evaporation, precursor decomposition, and particle nucleation occur within milliseconds. The high temperatures and short residence times characteristic of this process enable the formation of crystalline nanoparticles with high specific surface areas. However, the resulting materials often exhibit broad primary particle size distributions and a tendency towards aggregation, reflecting the strong coupling between flame dynamics, droplet evaporation, and particle growth.

Scaling flame‐based synthesis from laboratory to pilot or industrial scale presents nontrivial challenges. Increasing precursor concentration or liquid feed rate can enhance production rates, but often alters flame geometry, temperature profiles, and residence times. These changes strongly influence particle nucleation and growth, frequently leading to larger particle sizes or broader size distributions. In addition, increased liquid throughput typically requires larger spray droplets, which further promotes particle coarsening into the 50–100 nm regime. As a result, maintaining particle properties during scale‐up requires careful control of similarity parameters, including droplet size distribution, flame temperature gradients, and residence‐time histories.

Despite these challenges, flame spray synthesis offers distinct advantages for the preparation of perovskite catalysts. Conventional solid‐state or wet‐chemical routes to perovskites often require prolonged high‐temperature treatments that reduce surface area and limit catalytic performance. In contrast, FSS enables the direct formation of phase‐pure, crystalline perovskite nanoparticles with high surface areas, making it particularly attractive for catalytic applications. Accordingly, spray flame‐derived perovskites have been widely investigated for alkaline OER and other catalytic processes, where scalability and rapid material screening are essential [100, 101, 102].

In the recent years, intensive research on the correlation between perovskite type, raw material use and their catalytic activity for their application in electrolyzer technology has been performed since the replacement of noble raw material is driving the academic and industrial research. Bai et al. revealed compositional and structural changes on LaCoO_3_ and Ca‐doped LaCoO_3_ surfaces during OER and demonstrated how atomic‐scale compositional and structural details of electrocatalyst surfaces effect their activity and stability [103].

Beal et al. investigated bifunctional perovskite catalysts capable of catalyzing both the oxygen reduction reaction (ORR) and the oxygen evolution reaction (OER), synthesized via flame spray techniques. In their study, two distinct design strategies were explored. The first approach integrates ORR‐ and OER‐active transition metals, Mn and Co, within a single perovskite lattice. This was realized using the mixed‐metal perovskites Ba_0_._5_Sr_0_._5_Co_0_._8_Mn_0_._2_O_3_−δ (BSCM) and La_0_._5_Ba_0_._25_Sr_0_._25_Co_0_._5_Mn_0_._5_O_3_−δ (LBSCM) or Ir‐SrMnO_3_ [104]. The second strategy relies on a physical composite of two perovskite phases, combining an ORR‐active material, La_0_._4_Sr_0_._6_MnO_3_−δ (LSM), with an OER‐active perovskite, Ba_0_._5_Sr_0_._5_Co_0_._8_Fe_0_._2_O_3_−δ (BSCF), yielding a BSCF/LSM mixed catalyst. These systems highlight how compositional engineering and phase integration influence key physicochemical properties relevant to bifunctional oxygen electrocatalysis. The findings provide valuable comprehensions and design guidelines for the development of perovskite‐based bifunctional oxygen catalysts synthesized via scalable flame spray routes [103].

### 4.2 Sol‐Gel Synthesis

The sol–gel method is a versatile and efficient synthesis route for producing nano‐ and microstructured materials with precise control over particle size, morphology, surface area, and textural properties. Compared with many alternative synthesis strategies, sol–gel processing offers notable advantages including low cost, ease of implementation, and high compositional homogeneity, making it particularly attractive for catalyst development. Owing to its flexibility, this method is widely employed for both powder synthesis and the fabrication of thin films and coatings across diverse energy‐related applications [105].

A notable application of the sol–gel method is the stabilization of metastable perovskite phases that are inaccessible via conventional solid‐state routes. Using carefully controlled sol–gel synthesis conditions, including calcination temperature and citric acid content, a metastable strontium iridate phase, denoted γ‐SrIrO_3_ (γ‐SIO), was successfully obtained. This phase represents a third structural form of strontium iridate, distinct from the monoclinic 6H‐SrIrO_3_ (β‐SIO) and the pseudocubic 3C‐SrIrO_3_ (α‐SIO). Density functional theory calculations indicate that γ‐SIO possesses a higher formation energy (−724.60 kJ mol^−^^1^) than both β‐SIO (−768.12 kJ mol^−^^1^) and α‐SIO (−756.73 kJ mol^−^^1^), explaining why γ‐SIO cannot be synthesized by equilibrium solid‐state methods. Prior to electrochemical evaluation, γ‐SIO was exposed to acidic media, resulting in complete exchange of Sr^2^^+^ ions within its open framework with protons, yielding a protonated phase (H‐γ‐SIO). Remarkably, this ion‐exchange process proceeds without structural amorphization, preserving the crystalline framework while generating a highly hydroxylated surface. The resulting H‐γ‐SIO‐1 nanoparticles, with an average diameter of approximately 12 nm, exhibit outstanding acidic OER performance, delivering an overpotential of only 200 mV at 10 mA cm^−^^2^ (Figure 9a) and maintaining stable operation for over 1080 h (Figure 9b) [106]. During OER operation, H‐γ‐SIO undergoes a crystalline‐to‐crystalline transformation, forming ultrasmall (1–2 nm), (200)‐oriented rutile IrO_2_ nanoparticles anchored within the original framework. This transformation increases the density of catalytically active Ir sites while preserving structural integrity. The reaction proceeds predominantly via the adsorbate evolution mechanism (AEM), involving HO*, O*, and HOO* intermediates (Figure 9c), which contributes to the high intrinsic activity. The combination of reduced particle size, high stability number, and suppressed Ir dissolution ensures long‐term durability while maintaining a high population of active IrO_2_ sites.

**FIGURE 9:**
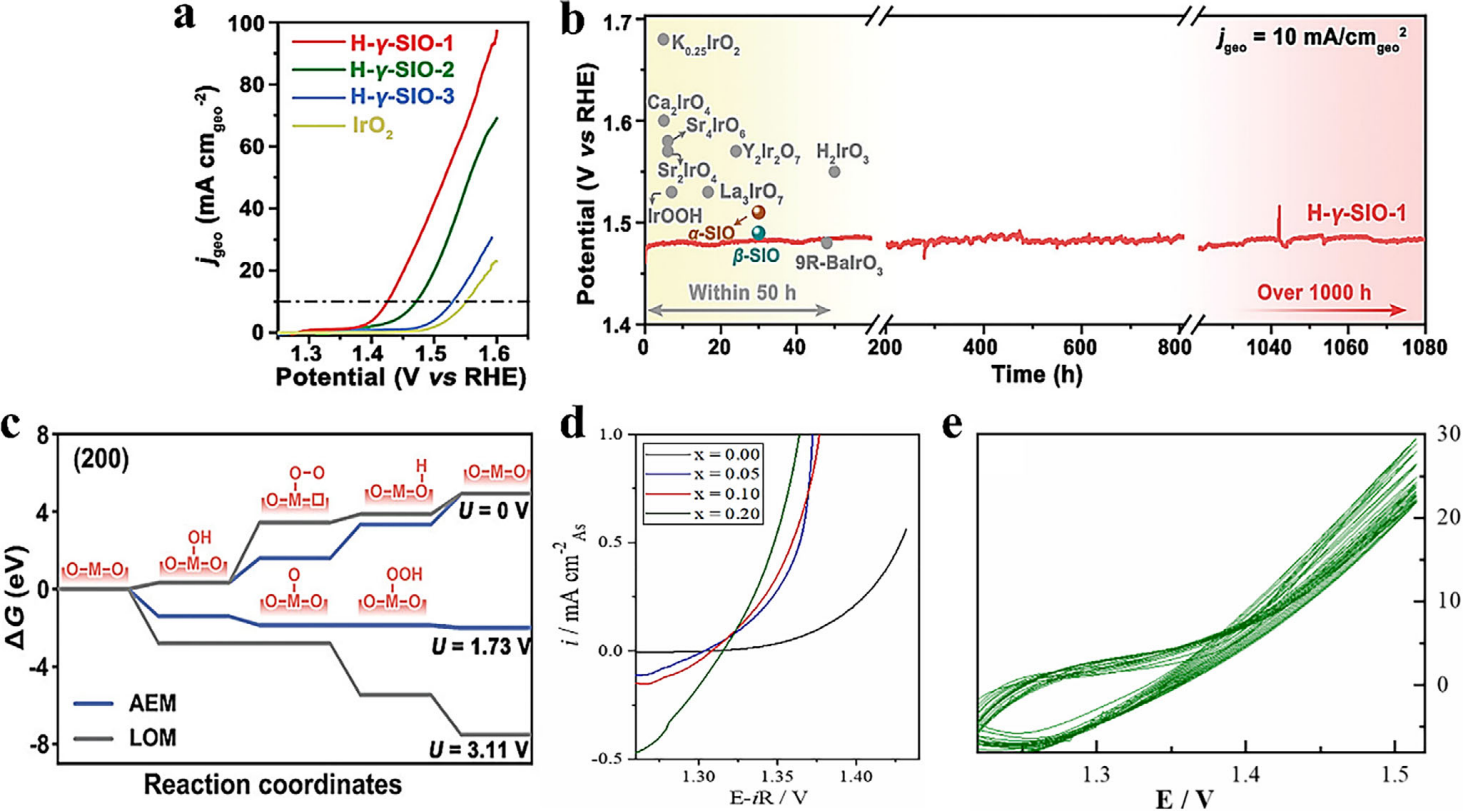
a) OER curves from different catalysts (deposited on 3 mm glassy carbon working electrode measured in 1 M HClO_4_ using LSV from 1.2 to 1.6 V versus RHE), (b) chronopotentiometric stability curve of different catalysts at 10 mA cm^−2^ (measured in 0.1 M H_2_ClO_4_), and (c) diagram illustrating free energy changes at four distinct stages under varying applied potentials for the (200) IrO_2_ surface, comparing AEM and LOM reaction pathway. Reproduced with permission [106]. Copyright 2021, MDPI. (d) Evolution of catalytic activity normalized to the mass‐specific surface area A_s_ curves of SrRuO_3_ (black line), Sr_0.95_K_0.05_RuO_3_ (blue line), Sr_0.90_K_0.10_RuO_3_ (red line), and Sr_0.80_K_0.20_RuO_3_ (green line) catalysts (deposited on glassy carbon working electrode measured at 1600 rpm rotation with RDE in O_2_‐saturated 0.1 M HClO_4_ using LSV from 1.2 to 1.5 V versus RHE at scanning rate of 10 mV s^−1^), and (e) evolution of OER over 20 cycles at 10 mA cm^−2^ for Sr_0.80_K_0.20_RuO_3_ (measured in O_2_‐saturated 0.1 M HClO_4_). Reproduced with permission [107]. Copyright 2023, John Wiley and Sons.

In addition to iridium‐based systems, sol–gel synthesis has been effectively applied to ruthenium‐based perovskites. Potassium‐doped SrRuO_3_ catalysts with well‐crystallized, phase‐pure structures were synthesized at relatively low calcination temperatures, enabling precise control over composition and homogeneous mixing of metal ions at the atomic scale. The optimized Sr_0_._80_K_0_._20_RuO_3_ catalyst exhibits the lowest overpotential among the investigated compositions (Figure 9d) and demonstrates enhanced durability, retaining approximately 75% of its initial activity after 20 consecutive cycles (Figure 9e) [107].

The improved OER performance of K‐doped SrRuO_3_ is attributed to multiple synergistic effects induced by A‐site substitution. Potassium incorporation increases the average Ru oxidation state from Ru^4^^+^ toward Ru^5^^+^, strengthening metal–oxygen interactions and facilitating faster charge transfer. Simultaneously, reduced RuO_6_ octahedral distortion enhances structural symmetry and orbital overlap, promoting more efficient electron transport. The sol–gel‐derived materials also exhibit increased mass‐specific surface areas, improving the accessibility of active sites. Importantly, K doping suppresses Sr leaching under acidic conditions, thereby preserving the perovskite framework and contributing to sustained catalytic stability.

Monoclinic SrIrO_3_ nanosheets (denoted M‐SrIrO_3_) were synthesized via a sol–gel route to achieve precise control over nanoscale morphology, phase uniformity, molecular‐level dispersion of metal precursors, and enhanced surface reactivity. This synthesis strategy enables accurate regulation of pH and calcination temperature during gel formation, yielding highly crystalline materials while simultaneously allowing subsequent sulfur incorporation through gas‐phase treatment. The resulting S‐doped M‐SrIrO_3_ catalyst exhibits a low overpotential of 228 mV at a current density of 10 mA cm^−^^2^ under acidic conditions (Figure 10a) and maintains stable operation for 20 h (Figure 10b) [74]. The enhanced acidic OER performance arises from the combined effects of crystal structure, morphology, surface reconstruction, and electronic modulation. The monoclinic SrIrO_3_ phase (Figure 10c), composed of face‐sharing IrO_6_ octahedra, promotes strong Ir–Ir metallic bonding, leading to improved electronic conductivity (Figure 10d). In addition, the nanosheet morphology (Figure 10e) provides a high density of exposed active sites, short ion and gas diffusion pathways, and improved interfacial contact with the electrolyte, collectively facilitating rapid reaction kinetics. During electrochemical operation, selective Sr leaching induces the formation of a thin amorphous Ir(O, S)_x_ surface layer that modifies the local coordination environment of Ir, increases oxygen vacancy concentration, and exposes additional catalytically active sites. Sulfur incorporation further tunes the electronic structure, reduces the free‐energy barrier for OER intermediates, and enhances charge transfer kinetics. Importantly, the in situ–formed Ir(O, S)_x_ layer acts as a protective shell that preserves the integrity of the monoclinic framework and suppresses bulk amorphization during prolonged operation. The reaction proceeds via the AEM, providing a more favorable OER pathway and contributing to the observed activity and durability under acidic conditions.

**FIGURE 10:**
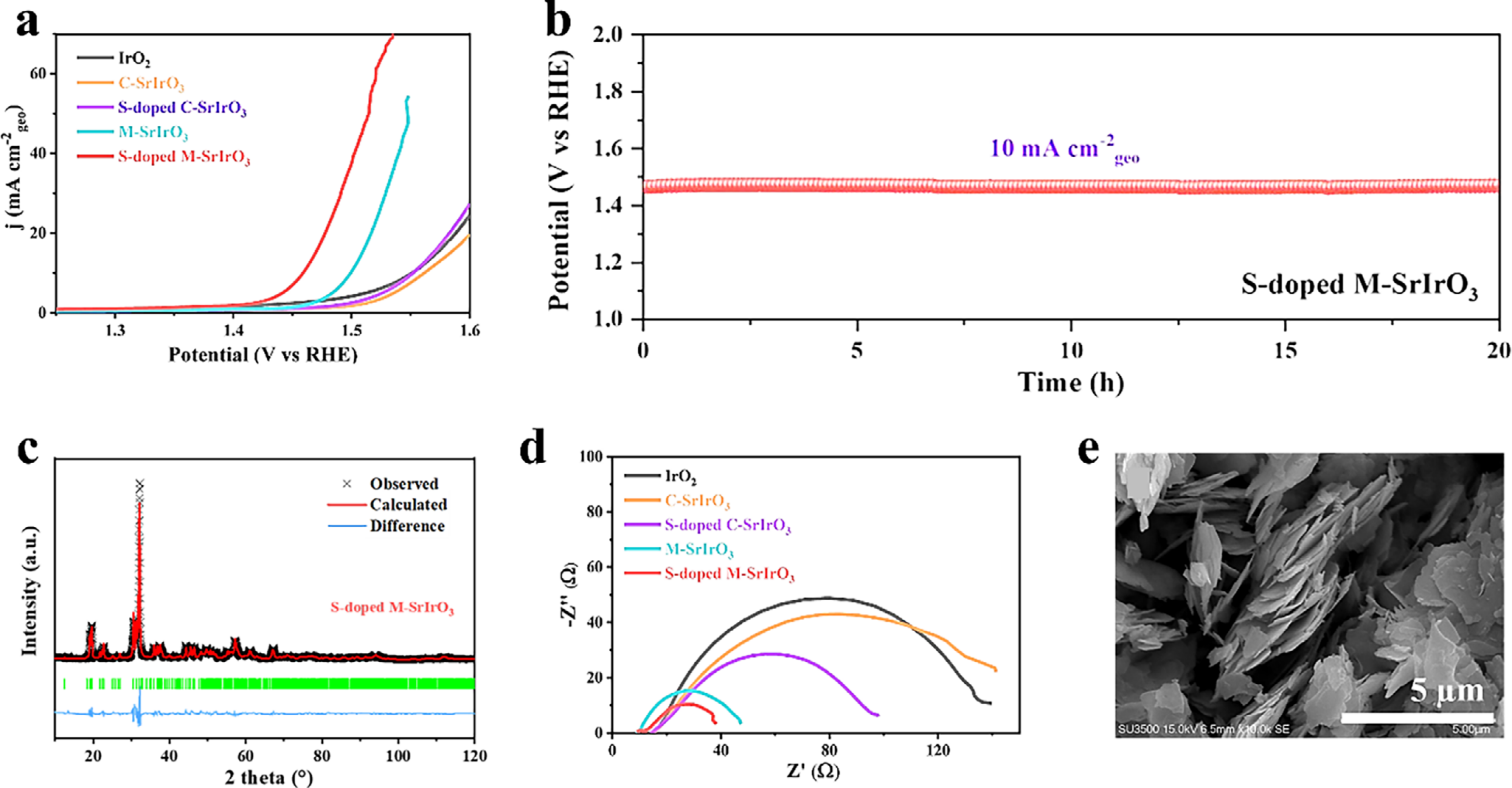
(a) OER curves from different catalysts (deposited on glassy carbon working electrode measured in O_2_‐saturated 0.5 M H_2_SO_4_ using LSV from 1.2 to 1.6 V versus RHE, iR‐drop compensated), (b) chronopotentiometric stability curve of S‐doped M‐SrIrO_3_ catalyst at 10 mA cm^−2^ (measured in O_2_‐saturated 0.5 M H_2_SO_4_), (c) XRD Rietveld refinement of S‐doped M‐SrIrO_3_, (d) EIS Nyquist plot of different catalysts (versus RHE), and (e) SEM image of S‐doped M‐SrIrO_3_. Reproduced with permission [74]. Copyright 2024, Elsevier.

### 4.3 Hydrothermal Synthesis

Hydrothermal synthesis is a widely used wet‐chemical method for catalyst preparation, in which precursor solutions are sealed in an autoclave and reacted under elevated temperature and pressure to promote controlled crystal growth. Although the technique is inherently time consuming and requires harsh operating conditions, and despite the limited in situ visibility into nucleation and growth processes, it remains attractive due to its simplicity, compositional flexibility, and ability to yield well‐defined crystalline materials [6, 108].

The method enables controlled crystallization and offers considerable flexibility for adjusting catalyst composition, defect chemistry, particle size, and morphology. Hydrothermally synthesized perovskites have, for example, been obtained as uniform nanorods, nanostructured doped oxides, and perovskite–carbon composites. The hydrothermal preparation of La(Co_0_._55_Mn_0_._45_)_0_._99_O_3_−δ produced highly crystalline nanorods with transition‐metal‐rich surfaces, while coupling with nitrogen‐doped reduced graphene oxide improved electrical connectivity and limited particle agglomeration [109]. Similarly, hydrothermally prepared Mn‐doped FeTiO_3_ demonstrated that the method can combine compositional modification with control of crystallite size and morphology, resulting in improved conductivity, a greater number of accessible active sites, and enhanced OER performance [110]. Hydrothermal synthesis has also been used to integrate perovskites with conductive supports, such as SrCeO_3_/rGO, to address the inherently limited conductivity and surface area of some perovskite oxides [111]. A promising PGM containing example is Ir‐doped calcium copper titanate, CaCu_3_Ti_4_O_12_, in which Ir was introduced at the Ti site. The framework provides high polarizability and abundant oxygen vacancies, whereas Ir incorporation enhances metal–oxygen covalency, lowers charge‐transfer resistance, and promotes the formation of surface IrO_x_ nanoclusters that act as the principal OER‐active species [99].

### 4.4 Solid‐State Synthesis

Solid‐state synthesis is a widely used and conceptually simple approach for preparing perovskite catalysts, typically involving intimate mixing of solid precursors followed by high‐temperature calcination to induce phase formation and crystallization. Although this method generally requires elevated temperatures and repeated grinding steps to ensure homogeneity, it remains attractive due to its robustness, compositional precision, and ability to produce highly crystalline bulk materials [112, 113].

Using this approach, bulk Ruddlesden–Popper layered perovskite Sr_2_IrO_4_ was synthesized through high‐temperature calcination of stoichiometric precursors, yielding a well‐ordered structure with a clearly defined IrO_6_ octahedral framework. The resulting catalyst (denoted HION) exhibits appreciable acidic OER activity, delivering an overpotential of 300 mV at 10 mA cm^−^^2^ in 0.1 M HClO_4_ (Figure 11a) and maintaining stable operation for 30 h and while exhibiting ∼0.15% Ir leaching (after 10 h; Figure 11b) [114]. The layered perovskite architecture provides a mechanically robust backbone that preserves structural integrity under acidic anodic polarization, while the Ir^4^^+^ electronic configuration creates a favorable electronic environment for OER. In addition, corner‐sharing IrO_6_ octahedra optimize the binding strength of oxygen intermediates, promoting efficient reaction kinetics. Surface hydroxyl groups further reduce the energetic barrier for oxygen evolution, while the nanosheet‐like morphology (Figure 11c) enhances charge transport, increases exposure of active sites, and improves adhesion to the electrode surface, mitigating passivation during operation.

**FIGURE 11:**
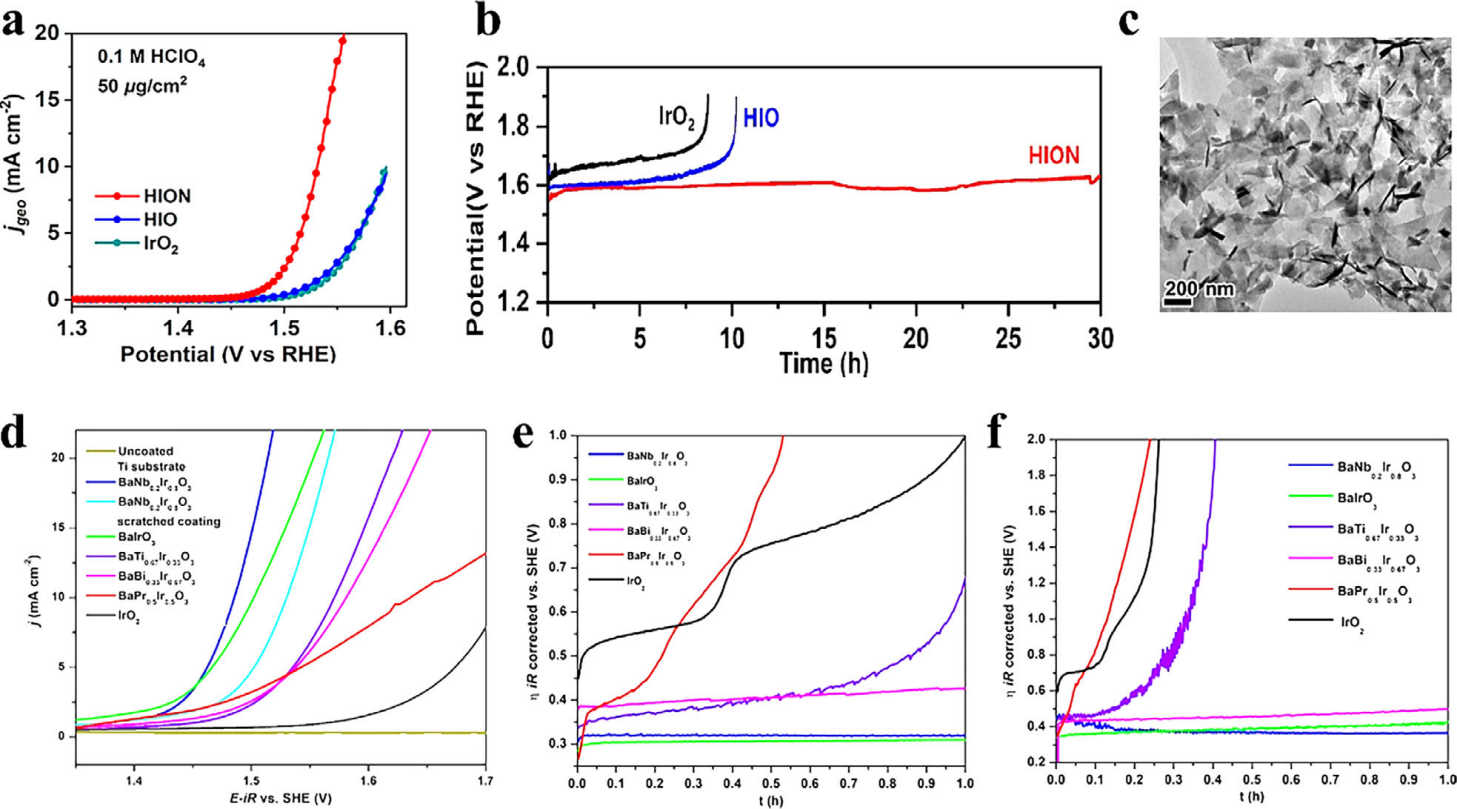
(a) OER curves from HIO, HION and IrO_2_ catalysts (catalyst loading 50 µg cm^−2^ deposited on glassy carbon working electrode measured in 0.1 M HClO_4_ using LSV from 1.3 to 1.6 V versus RHE), (b) chronopotentiometric stability curve of SrIrO_3_, HION and IrO_2_ at 10 mA cm^−2^ (measured in 0.1 M HClO_4_), and (c) TEM image of HION. Reproduced with permission [114]. Copyright 2021, American Chemical Society. (d) OER curves from different catalysts (deposited on 1 cm^−2^ titanium working electrode measured in 1.7 M H_2_SO_4_ at 40°C using LSV from 1.2 to 1.9 V versus RHE), galvanostatic stability curve of different catalysts (measured in 1.7 M H_2_SO_4_ at 40°C) at (e) 100 mA cm^−2^ and (f) 500 mA cm^−2^. Reproduced with permission [97]. Copyright 2022, American Chemical Society.

Solid‐state synthesis has also been applied to more compositionally complex perovskites aimed at tuning activity and stability through B‐site substitution. A series of BaM_x_Ir_1__−__x_O_3_ perovskite catalysts were prepared via sequential mixing, ball milling, high‐temperature calcination, and controlled cooling, resulting in homogeneous elemental dispersion and highly crystalline phases with precise compositional control. Among these materials, BaNb_0_._2_Ir_0_._8_O_3_ exhibited notable acidic OER performance, achieving overpotentials of 250 and 340 mV at current densities of 100 and 500 A m^−^^2^, respectively (Figure 11d), and maintaining stability for 1 h in H_2_SO_4_ electrolyte (Figure 11e,f) [97].

The improved performance of BaNb_0_._2_Ir_0_._8_O_3_ arises from the synergistic effects of structural and electronic modulation. The perovskite lattice provides an optimized coordination environment that enhances the intrinsic activity of Ir active sites, while the formation of a stable rhombohedral structure suppresses rapid degradation under acidic conditions. Importantly, minimal sulfate adsorption on the catalyst surface preserves active Ir sites, and Nb incorporation stabilizes the perovskite framework while improving electronic conductivity. Together, these effects demonstrate that, despite its limitations in surface area control, solid‐state synthesis remains a viable route for producing structurally robust perovskite catalysts for acidic OER applications.

### 4.5 Pulsed Laser Deposition

Pulsed laser deposition (PLD) enables precise control over stoichiometry, thickness, and exposed crystal facets in multicomponent oxide thin films, making it a powerful tool for fundamental studies of perovskite electrocatalysts. In PLD, laser ablation of a solid target generates a transient plasma plume that deposits material onto a substrate, where nucleation and film growth occur under carefully controlled conditions [115]. This high level of control makes PLD particularly valuable for preparing well‐defined model systems to probe intrinsic activity, surface reconstruction, and degradation mechanisms. PLD‐grown thin films have been widely employed to investigate the apparent stability of perovskite catalysts under acidic OER conditions. For example, SrIrO_3_ thin films synthesized by PLD exhibited stable operation for approximately 6 h at ∼1.70 V versus RHE while delivering a current density of 10 mA cm^−^^2^. However, high‐resolution transmission electron microscopy revealed a pronounced reduction in film thickness from ∼40 nm to ∼18 nm after operation, indicating substantial material loss despite the seemingly stable electrochemical performance [5, 116]. This behavior reflects a layer‐by‐layer dissolution mechanism, in which Sr leaching from the bulk leads to the formation of an IrO_x_‐rich surface layer that progressively dissolves under anodic bias. Importantly, the rate of degradation was strongly dependent on the applied potential, demonstrating that conventional stability metrics based solely on current retention can significantly overestimate true material durability.

Although PLD offers control and reproducibility, its application in the synthesis of perovskite‐based acidic OER catalysts remains limited. The technique is inherently unsuitable for large‐scale catalyst production due to low deposition rates, limited throughput, and high operational costs [35, 117]. As such, PLD is best viewed as a complementary approach for mechanistic investigations and benchmarking studies, rather than a practical route for manufacturing acidic OER catalysts intended for device‐level deployment. Conclusively, the discussed synthesis approached are listed in Table 1, showing their key features, limitations and relevance.

**TABLE 1:** The major synthesis routes for perovskite based acidic OER catalysts.

Synthesis method	Key features	Advantages for acidic OER	Limitations/challenges	Scalability & relevance
Solid‐state synthesis	High‐temperature calcination of mixed precursors; bulk crystalline phases	High phase purity; thermodynamically stable structures; suitable for fundamental structure–activity studies	Large particle size; low surface area; limited control over morphology; high energy input	High scalability; industrially established, but requires post‐processing for high activity
Sol–gel synthesis	Molecular‐level mixing of metal precursors via chelation and gelation	Excellent compositional control; tunable particle size and morphology; enables metastable phases and controlled doping	Organic residues possible; multi‐step processing; batch‐to‐batch variability	Medium scalability; widely used in laboratories and adaptable to scale‐up
Spray‐flame synthesis	Rapid combustion of aerosolized precursors at high temperature	Produces high‐surface‐area, crystalline nanoparticles; short residence time limits phase segregation	Limited compositional flexibility; challenging defect control; acidic OER still underexplored	High scalability; promising for industrial catalyst production
Hydrothermal synthesis	Crystallization in sealed autoclaves under high temperature and pressure	Precise morphology control; high defect density; uniform dopant distribution	Long synthesis times; limited phase space; safety concerns at scale	Low–medium scalability; mainly suitable for exploratory studies
Electrospinning	Formation of 1D nanofibers followed by calcination	High surface area; continuous electron pathways; enhanced mass transport	Complex processing; fiber breakage during calcination; limited compositional range	Medium scalability; promising for structured electrodes
Pulsed laser deposition (PLD)	Laser ablation of targets to form thin films with controlled stoichiometry	Atomic‐scale control; ideal for mechanistic and stability studies	High cost; low throughput; not suitable for bulk catalyst production	Very low scalability; model‐system technique only
Spray coating/infiltration (for MEA)	Deposition of catalyst inks onto membranes or porous transport layers	Direct relevance to PEMWE; enables low Ir loadings and realistic testing	Ink formulation complexity; ionomer–catalyst interactions	High relevance for device integration; industrially viable

## 5 Surface and Morphological Requirements

The previous sections established which perovskite compositions exhibit promising activity under acidic OER and how these materials can be synthesized. However, the intrinsic properties of a catalyst alone do not determine its electrochemical performance. Surface area, particle size, porosity, morphology, and the distribution of active sites strongly influence catalyst utilization, mass transport, and long‐term durability

Surface structure and morphology play a decisive role in governing the activity and durability of perovskite‐based OER catalysts. Nanostructured morphologies, including porous architectures, nanoparticles, nanorods, nanospheres, nanotubes, and fibrous networks, enhance catalytic performance by increasing the density of exposed active sites, shortening ion and gas diffusion pathways, and facilitating efficient transport of reactants and products [40, 118].

A representative example is the SrTi_0_._67_Ir_0_._33_O_3_ (Ir–STO) perovskite, designed to reduce iridium content while maximizing mass activity. Compared with IrO_2_, Ir–STO contains 57 wt.‐% less Ir yet delivers nearly an order‐of‐magnitude higher activity. The catalyst achieves an overpotential of 295 mV at a current density of 43 mA cm^−^^2^ (Figure 12a) and maintains stable operation for 20 h at both 10 and 30 mA cm^−^^2^ under acidic conditions (Figure 12b,c) [119]. Electron microscopy and BET analysis reveal an interconnected nanoparticle network with an average particle size of approximately 50 nm and a specific surface area of 45 m^2^ g^−^^1^ (Figure 12d,e). High‐resolution TEM further shows that the dominant exposed facet corresponds to the {001} surface, as indicated by the 45° angle between the (200) and (110) lattice planes (Figure 12f). Density functional theory calculations suggest that this facet is particularly active because Ti sites on the {001} surface are electronically activated by adjacent Ir dopants, enabling enhanced catalytic turnover. The interconnected nanoparticle morphology thus maximizes the availability of highly active surface planes, resulting in improved OER efficiency.

**FIGURE 12:**
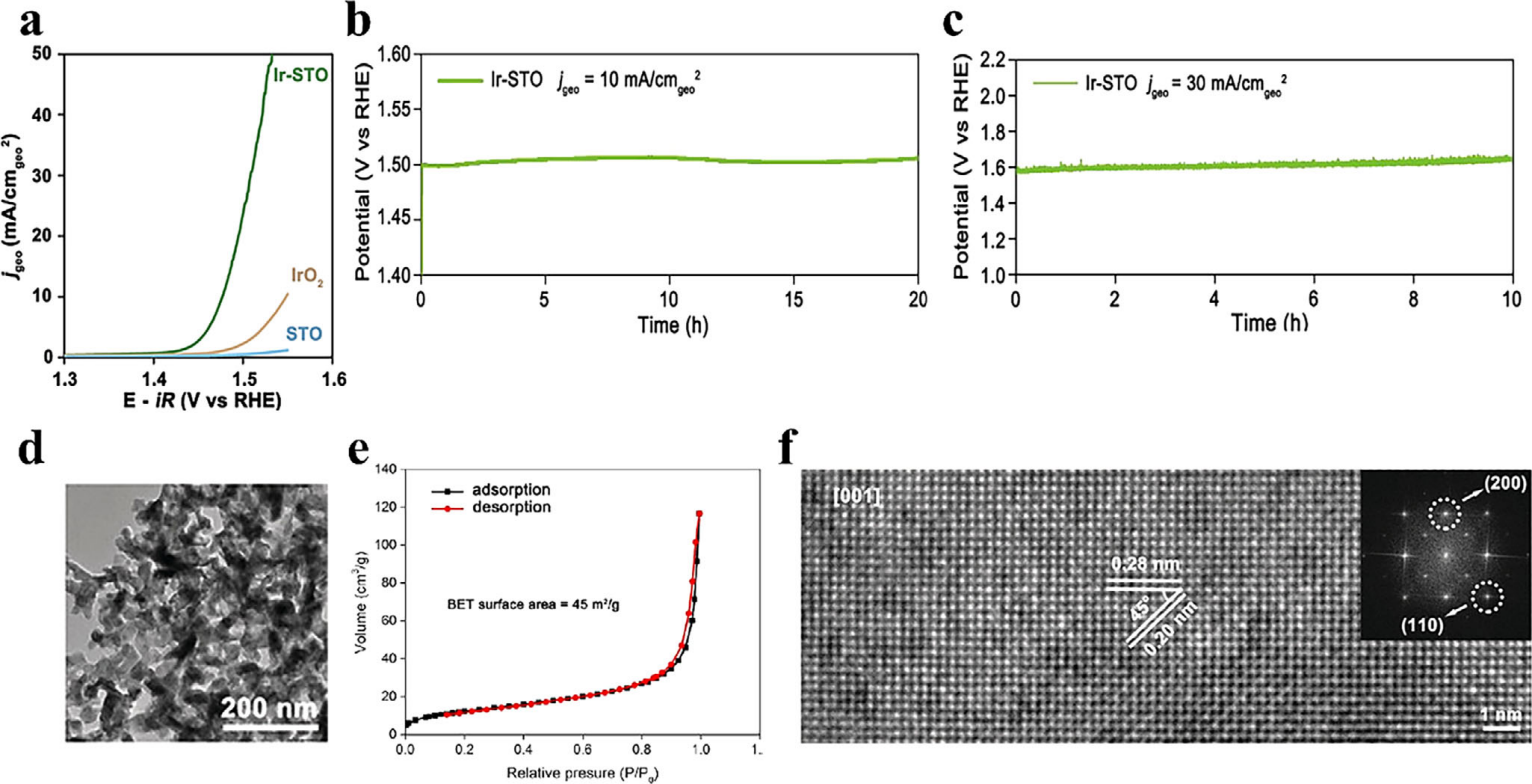
(a) OER curves from SrTi_0.67_Ir_0.33_O_3_ (Ir‐STO), SrTiO_3_ (STO) and IrO_2_ catalysts (deposited on 3 mm glassy carbon working electrode measured in 0.5 M HClO_4_ with N_2_‐bubbling during measurement using LSV from 1.3 to 1.55 V versus RHEw with 5 mV s^−1^ scan rate, iR‐drop compensated), (b) chronopotentiometric stability curve (measured in 0.5 M HClO_4_ with N_2_‐bubbling) of Ir‐STO at (b) 10 mA cm^−2^ and (c) 30 mA cm^−2^, (d) TEM image of Ir‐STO, (e) N_2_ adsorption–desorption isotherms of Ir‐STO, and (f) high resolution TEM (HRTEM) image of Ir‐STO with inset showing fast Fourier transform image. Reproduced with permission [120]. Copyright 2025, Elsevier.

Beyond facet engineering, strong metal–support interactions and phase heterogeneity offer additional strategies to reconcile activity and stability. While crystalline materials typically exhibit high structural robustness but limited activity, amorphous phases often deliver superior activity at the expense of durability. Combining these characteristics within a single heterostructured catalyst can therefore provide synergistic benefits. In this context, a crystalline–amorphous La_0_._6_Ca_0_._4_Fe_0_._8_Ni_0_._2_O_3_ (LCFN) hollow porous nanotube framework supporting IrOx nanoparticles was developed (Figure 13a). The resulting composite catalyst, denoted IrOx@LCFN‐96 (mass ratio 2:1), exhibits a low overpotential of 143 mV at 10 mA cm^−^^2^ in strongly acidic media (pH = 1) and maintains stable operation for 20 h (Figure 13b,c) [121]. In this system, the amorphous regions supply a high density of unsaturated coordination sites that promote activity, while the crystalline domains provide mechanical and chemical stability. The hollow nanotube architecture further enhances mass transport and ensures uniform dispersion of IrOx nanoparticles. Notably, the participation of the lattice oxygen mechanism (LOM) lowers the energetic barrier for OER by bypassing conventional rate‐determining steps, contributing to the observed high activity.

**FIGURE 13:**
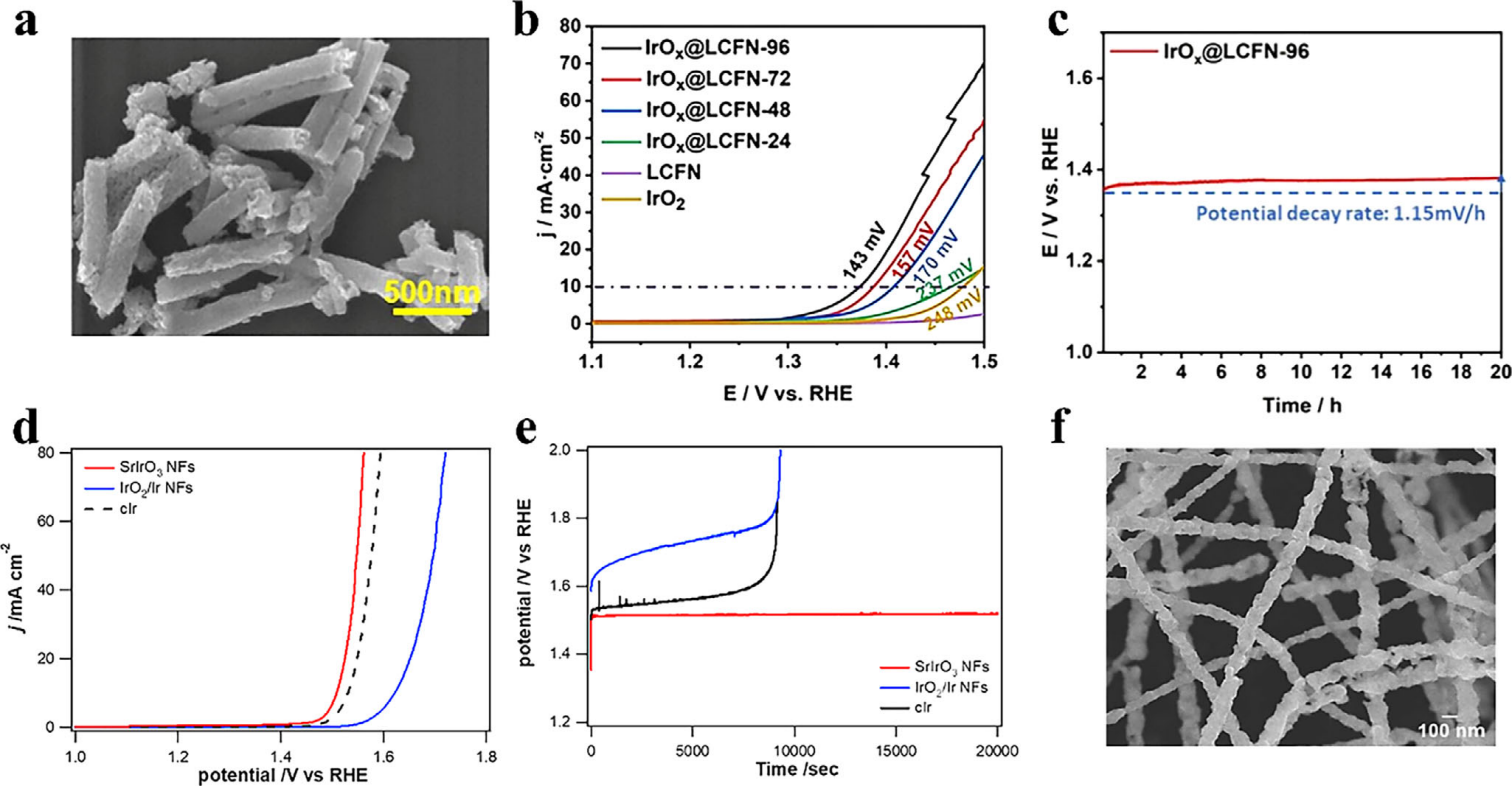
(a) SEM image of IrO_x_@LCFN‐96 (IrO_x_ at La_0.6_Ca_0.4_Fe_0.8_Ni_0.2_O_3_ (LCFN) synthesized from 96 mg IrCl_3_ and 48 mg LCFN), (b) OER curves from different catalysts (deposited on 3 mm glassy carbon RDE working electrode measured in 1 M H_2_SO_4_ using LSV from 1.3 to 1.55 V versus RHE with 5 mV s^−1^ scan rate, iR‐drop compensated), (c) chronopotentiometric stability curve (measured in 0.1 M H_2_SO_4_) of IrO_x_@LCFN‐96 at 10 mA cm^−2^ and 1600 rpm. Reused with consent ^100^. (d) OER curves from commercial Ir (cIr), SrIrO_3_ nanofibers (NFs) and IrO_2_/Ir NFs (deposited on 3 mm glassy carbon RDE working electrode measured in 0.5 M H_2_SO_4_ using LSV from 1.0 to 1.8 V versus RHE with 10 mV s^−1^ scan rate and 1600 rpm), (e) chronopotentiometric stability curve (measured in 0.5 M H_2_SO_4_) of cIr, SrIrO_3_ NFs and IrO_2_/Ir NFs at 10 mA cm^−2^ and 1600 rpm, and (f) SEM image of SrIrO_3_ NFs. Reproduced with permission [121]. 2019, American Chemical Society.

Electrospinning offers another effective route to overcome the limitations of conventional solid‐state synthesis, which often yields large particles with low surface areas. Using this approach, single‐phase perovskite SrIrO_3_ nanofibers were fabricated, forming a continuous fibrous network with nanoscale dimensions. The resulting catalyst achieves an overpotential of 280 mV at 10 mA cm^−^^2^ (Figure 13d) and exhibits stable operation for 20 000 s in acidic electrolyte (Figure 13e) [122]. The nanofiber morphology (Figure 13f) provides a large accessible surface area and abundant active sites, while the homogeneous perovskite structure ensures structural stability despite partial Sr leaching. Loosely arranged, corner‐sharing IrO_6_ octahedra and low charge‐transfer resistance further facilitate rapid electron transport, enabling sustained catalytic activity under acidic OER conditions.

## 6 Integration in Membrane Electrode Assemblies

Many catalysts exhibiting excellent performance in three‐electrode measurements fail to reproduce these results under realistic PEM electrolyzer conditions because of limitations related to catalyst‐layer architecture, ionomer interactions, transport phenomena, and interfacial resistances. This section therefore addresses how the promising catalyst concepts discussed previously translate into practical device performance and identifies the remaining challenges for their implementation in PEM water electrolyzers.

Within PEMWE cells, the membrane electrode assembly (MEA) constitutes the functional core, integrating multiple transport and electrochemical processes into a single unit. The MEA comprises key components: the cathodic catalyst layer (CCL), the anodic catalyst layer (ACL), the proton exchange membrane (PEM), the gas diffusion layer (GDL), and the porous transport layer (PTL). The ACL drives the oxygen evolution reaction (OER) and is directly interfaced with the PEM, which selectively transports protons from the anode to the cathode while blocking electrons and gas crossover. Simultaneously, the ACL is in touch with the electrically conductive and porous PTL which ensures efficient water delivery and enables the rapid removal of oxygen bubbles generated during OER. Well‐designed PTL prevent mass‐transport limitations, especially at high current densities [123, 124, 125]. However, not only the cell setup and the catalyst material as well as its loading, but especially the ACL design affects the PEMWE performance. Ionomer content and ACL porosity being relevant factors improving proton conduction to connect membrane distant catalyst particles as well as enhancing media transport within the catalyst layer (water feed and O_2_ bubble removal) [126]. Contradictory, ionomer material in the ACL can have negative effects on electrical conductivity and/or on media transport. Depending on the catalyst type and cell architecture, therefore, an optimal microstructure and ionomer content have to be investigated when designing an ACL. A further effect on ACL performance results from the catalyst layer preparation technique, for example, direct spray or slot die coating, decal transfer processes or sputtering (especially, if OER catalyst material is decorated on top of a PTL instead on the PEM).

Despite the technological maturity of PEMWE, large‐scale deployment for which high performance and long‐term stability is relevant remains constrained by the reliance on iridium‐based catalysts, which are typically used at loadings of 1–3 mg_Ir_ cm^−^^2^ at the anode [127, 128] y]. Given the scarcity and cost of iridium, reducing Ir loading without compromising performance or durability is a central challenge. Importantly, lowering Ir content often reveals that electrochemical activity and stability in MEAs are governed not by the pristine catalyst structure, but by dynamic surface reconstruction processes that occur under realistic operating conditions.

A compelling demonstration of this principle is given by a series of double perovskite catalysts, Sr_2_MIrO_6_ (M = Ca, Zn, Mg). Among these materials, Sr_2_CaIrO_6_ exhibits particularly strong performance, achieving a cell voltage of 2.40 V at 6 A cm^−^^2^ and 80°C (Figure 14a), while maintaining stable operation for 450 h in PEMWE (Figure 14b) at an Ir loading of 0.4 mg_Ir_ cm^−^^2^ and an ionomer loading of 25 to 30 wt.‐% [129]. Rapid Ca^2^^+^ and Sr^2^^+^ leaching induces the formation of a porous, reconstructed surface enriched in highly active Ir sites. Concomitantly, short‐range ordered, edge‐sharing IrO_6_ octahedra emerge, reducing Ir–Ir distances and accelerating reaction kinetics. The stabilization of high‐valent Ir species (Ir^5^^+^/Ir^6^^+^), together with the occupation of lattice voids by hydronium (H_3_O^+^) ions, yields a mechanically and chemically robust framework capable of sustaining extreme current densities [129].

**FIGURE 14:**
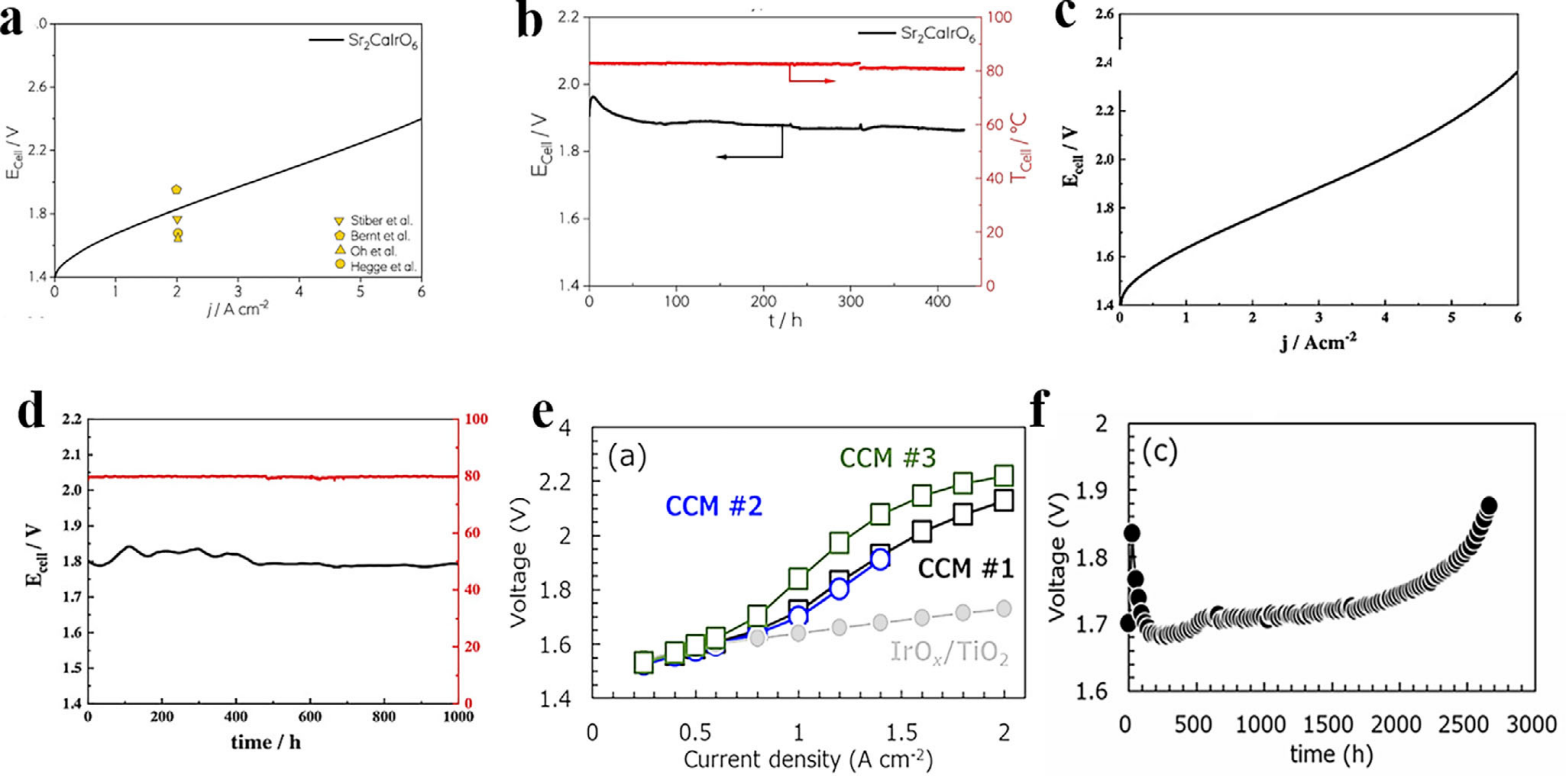
PEMWE measurement with commercial Pt/C cathode (0.4 mg_Pt_ cm^−2^) and Sr_2_CaIrO_6_ anode (0.4 mg_Ir_ cm^−2^) at ambient pressure and 80°C including literature values [130, 131, 132, 133] using Nafion N212 based PEMWE CCMs. (a) Galvanostatic measured cell potential versus current density (up to 6 A cm^−2^ (overload) according to the JRC EU‐harmonized) and (b) Cell potential and cell temperature at constant 2 A cm^−2^ over 450 h. Reproduced with permission [129]. Copyright 2023, John Wiley and Sons. PEMWE measurement with commercial Pt/C cathode (0.4 mg_Pt_ cm^−2^) and Sr_2_CaIrO_6_ anode (0.2 mg_Ir_ cm^−2^) at ambient pressure and 80°C. (c) Galvanostatic measured cell potential versus current density (up to 6 A cm^−2^ (overload) according to the JRC EU‐harmonized) and (d) Cell potential and cell temperature at constant 2 A cm^−2^ over 1000 h. Reproduced with permission [127]. Copyright 2025, John Wiley and Sons. (e) Potential versus current density for commercial IrO_x_/TiO_2_ CCM (anode loading 0.86 mg_Ir_ cm^−2^) and three SrTi_0.67_Ir_0.33_O_3_ CCMs (CCM‐#1, CCM‐#2 anode loading 0.17 mg_Ir_ cm^−2^ and CCM‐#3 anode loading 0.26 mg_Ir_ cm^−2^) all with Pt/C cathode (loading 0.5 mg_Pt_ cm^−2^) at 60°C and (f) stability curve for SrTi_0.67_Ir_0.33_O_3_ CCM‐#2 at 1 A cm^−2^ (at 60°C). Reproduced with permission [134]. Copyright 2024, Elsevier.

The critical role of leaching and structural rearrangement under MEA conditions is further illustrated by the Sr_2_CaIrO_6_ perovskite catalyst, integrated as an anode material at a 50% lower Ir loading of approximately

0.2 mg_Ir_ cm^−^^2^ and an ionomer loading of 20 to 30 wt.‐%. When tested in a laboratory PEMWE test cell, the material configuration including this catalyst as well as a standard Pt on carbon black catalyst for the CCL (with a loading of 0.4 mg cm^−2^) delivers a cell voltage of about 1.78 V at 2.0 A cm^−^^2^ (Figure 14c) and maintains stable operation for over 1000 h (Figure 14d) [115]. Under anodic polarization, selective leaching of Sr^2^^+^ and Ca^2^^+^ triggers extensive surface reconstruction, forming a porous, sponge‐like Ir–O–H phase dominated by IrOx and IrOOH species. This reconstructed surface layer constitutes the true catalytically active phase, offering a high density of accessible active sites and enhanced intrinsic activity. Elevated Ir oxidation states (Ir^5^^+^/Ir^6^^+^) further promote OER kinetics, while the formation of large, low‐tortuosity catalyst particles (1–3 µm thick) ensures efficient ion and electron transport within the catalyst layer. The mechanically robust reconstructed particles resist detachment and degradation, and sustained electronic connectivity to the PTL compensates for partial ionomer‐induced conductivity losses over time. In addition, in both described studies about Sr_2_CaIrO_6_ based ACLs the use of an about 50 µm thin membrane (Nafion 212) and improved interfacial contact contribute to reduced high‐frequency resistance and overall cell efficiency.

While numerous studies report high activity at reduced Ir loadings under moderate conditions, fewer have demonstrated durability under prolonged PEMWE operation. In this context, an ACL composed of the perovskite SrTi_0_._67_Ir_0_._33_O_3_, operating at an ultra‐low Ir loading of 0.17 mg_Ir_ cm^−^^2^ and an ionomer loading of 20 wt.‐%, provides a stringent benchmark. With such an ACL cell voltages of 1.72 V at 1.0 A cm^−^^2^ and 2.13 V at 2.0 A cm^−^^2^ (Figure 14e) were achieved, while sustaining stable operation for 2660 h at a constant current density of 1.0 A cm^−^^2^ and 60°C in a PEMWE device (Figure 14f) [116]. In this system, the spontaneous dispersion of Ir species onto a TiO_2_‐rich support maximizes Ir utilization and exposes a large population of active sites. The hierarchically porous TiO_2_ framework enhances electrolyte penetration and mass transport while providing strong anchoring sites for isolated Ir atoms. Robust Ir–O–Ti bonding suppresses Ir dissolution and aggregation, stabilizing catalytically active Ir^4^^+^ species and enabling favorable adsorption–desorption energetics for OER intermediates. The presented results have been obtained with the aid of 50 µm thin Nafion 212 membranes.

These examples demonstrate that the successful integration of perovskite‐based catalysts into MEAs relies on embracing, rather than suppressing dynamic reconstruction under operating conditions. Perovskite‐derived catalysts function not as static materials, but as precursors to highly active and durable IrOx‐based surface phases, supported by structurally resilient backbones that ensure electronic connectivity and mechanical stability. As such, MEA‐level evaluation is indispensable for assessing the true viability of perovskite catalysts.

## 7 In Situ and Operando Characterization of Perovskite Catalysts Under Acidic OER Conditions

The next logical question is whether these advances translate into meaningful improvements under realistic operating conditions. This section therefore moves beyond fundamental material properties to evaluate catalyst performance using device‐relevant metrics, highlighting the differences between laboratory‐scale electrochemical measurements and practical electrolyzer operation.

Recent studies have increasingly demonstrated that catalysts undergo significant reconstruction during acidic OER, with dynamic surface chemistry influencing the entire process, thereby challenging traditional mechanistic concepts predicated on the assumption of a well‐defined electrode–electrolyte interface. Even when ex‐situ electrochemical measurements indicate stable performance, the catalyst surface often undergoes dynamic reconstruction, changes in oxidation state, and reorganization of the metal–oxygen framework. Furthermore, protocols for the preparation of catalyst layers for RDE measurements, for example, differ from those for ACLs in MEAs, as described in the previous section. For RDE preparation, the catalyst layer has to adhere to the substrate at disk rotation speeds that often exceed 1000 rpm in order to efficiently remove the gas bubbles produced. This requires different specifications such as different catalyst‐to‐ionomer ratios as well as different catalyst coating techniques such as drop coating, compared with those used for ACLs for in situ testing. Finally, ex situ testing is usually performed up to a maximum electrode size of 1 cm^2^ while commercial laboratory PEMWE test cells typically contain electrodes with sizes of 4 to 25 cm^2^ or even beyond [135, 136, 137, 138, 139, 140]. As a result, ex‐situ characterization, such as stand‐alone RDE measurements, is insufficient to capture the true nature of active sites or degradation pathways under operating conditions [141, 142, 143].

In situ and operando characterization techniques have therefore become indispensable for understanding the structure–activity–stability relationships of perovskite catalysts during acidic OER. These approaches enable real‐time monitoring of electronic structure, coordination environment, and phase evolution while the catalyst is actively operating, providing insights that cannot be obtained from post‐mortem analysis [144, 145].

### 7.1 Operando Spectroscopic Techniques: XAS and Related Methods

Among in situ techniques, X‐ray absorption spectroscopy (XAS) has emerged as one of the most powerful tools for probing perovskite‐based catalysts under acidic OER conditions. Operando XAS enables real‐time tracking of changes in oxidation state, metal–oxygen bond lengths, and local coordination geometry of catalytically active centers during electrochemical operation, providing in that are inaccessible through ex situ characterization.

Across multiple operando XAS studies on Ir‐ and Ru‐based perovskite catalysts, a consistent picture has emerged in which the catalytically active state differs markedly from the pristine crystalline structure, particularly under anodic polarization relevant to acidic OER. For example, operando Ir L_3_‐edge XAS measurements on Zn‐doped SrIrO_3_ (SIZO) revealed a progressive increase in the Ir oxidation state under applied bias, associated with surface deprotonation and activation processes that were not detectable by ex situ analysis [96]. Similar behavior has been observed in Ca‐ and Sr‐substituted iridate perovskites, where dynamic changes in Ir valence and coordination accompany OER operation [98, 128]. In several systems, operando XAS and EXAFS analyses indicate partial loss of long‐range order near the surface and the formation of amorphous IrO_x_‐ or IrOOH‐like layers during OER, while the bulk perovskite framework remains largely intact [98, 128]. These reconstructed surface layers are often identified as the catalytically active phase, emphasizing that high activity in acidic OER is frequently linked to controlled surface amorphization rather than preservation of the pristine lattice.

Importantly, operando XAS studies have shown that surface reconstruction can proceed even when electrochemical measurements such as chronoamperometry suggest stable performance. In the case of SIZO, extended electrochemical cycling led to significant electronic and structural evolution, including a metal–insulator transition attributed to defect formation and amorphization, despite apparently stable OER currents [96]. Likewise, operando XAS of Sr_2_CaIrO_6_ revealed rapid changes in Ir oxidation state and coordination geometry during OER, consistent with a phase transition rather than slow, gradual surface rebuilding [128].

While operando spectroscopic techniques such as XAS provide invaluable mechanistic insights into the dynamic nature of perovskite catalysts under acidic OER conditions, most studies are conducted in half‐cell configurations and at relatively modest current densities. As a result, they do not fully capture the complex degradation pathways, transport limitations, and mechanical stresses encountered during electrolyzer operation, underscoring the need to complement spectroscopic operando studies with in situ electrolysis experiments under device‐relevant conditions.

### 7.2 In Situ Electrolysis Under Device‐Relevant Conditions

In contrast to operando spectroscopic studies performed in half‐cell configurations, in situ electrolysis experiments conducted under device‐relevant conditions remain extremely scarce for perovskite‐based acidic OER catalysts. These experiments, are typically carried out using PEWME test cells in which MEAs are integrated. When testing catalyst layers in a test cell, the material setup (PTL, CCM, and GDL) and the ACL design are not the noly important factors. Uniform contact between the different components of the MEA as well as homogeneous cell clamping and waste heat removal are crucial for an optimal in situ PEMWE testing [137]. Then, the operation of an MEA within a full cell provides direct insight into catalyst behavior under realistic operating parameters, including high current densities, elevated temperatures, strong proton flux, and complex catalyst–ionomer–support interactions.

A recent example of meaningful in situ electrolysis is provided by the study on Sr_2_CaIrO_6_‐based anodes operated in commercial laboratory PEMWE cells [126]. In this work, polarization curves and long‐term galvanostatic measurements were recorded directly during electrolyzer operation at current densities up to 2.0 A cm^−^^2^ and a temperature of 80°C. In situ electrolysis revealed that controlled leaching of Sr^2^^+^ and Ca^2^^+^ during operation induces surface reconstruction into a sponge‐like, amorphous Ir–O–H (IrO_x_/IrOOH) phase, which serves as the active state for acidic OER. Crucially, this reconstructed catalyst layer exhibited remarkable stability over 1000 h of continuous operation, a behavior that cannot be inferred from half‐cell testing alone. Further insight into reconstruction‐driven performance under realistic conditions is provided by Sr_2_MIrO_6_ (M = Ca, Zn, Mg) perovskite anodes, where in situ electrolysis revealed that rapid A‐site leaching during PEMWE operation leads to the formation of short‐range‐ordered, edge‐sharing IrO_6_ clusters that sustain high activity at current densities of up to 6 A cm^−^^2^ [128]. In both works, test cells with an active cell area of 4 cm^2^ were used for in situ testing.

Similarly, device‐level in situ electrolysis studies on SrTi_0_._67_Ir_0_._33_O_3_ (CCM‐type anodes) demonstrated that extremely low Ir loadings (≈0.17 mgIr cm^−^^2^) can be sustained under prolonged PEMWE operation [133]. In this case, a test cell with geometric electrode area of 5 cm² was utilized. In situ polarization curves and durability testing at 60°C and 1.0 A cm^−^^2^ revealed stable operation for more than 2600 h. The in situ data showed that the automatic dispersion of Ir onto a TiO_2_‐rich support during operation plays a decisive role in maintaining catalytic activity and suppressing Ir dissolution. Such dynamic redistribution and stabilization of active Ir species cannot be resolved by operando spectroscopy alone.

This section has reported studies that represent successful perovskite OER catalyst development for PEMWE, including both ex situ analyses and full‐cell testing. However, the in situ results presented were obtained in rather small‐scale laboratory test cells with electrode geometries below 10 cm², which do not represent the industrial‐scale—a common issue in academic catalyst research [146]. Actually, the upscaling perovskite‐based catalyst layers for acidic OER and their analysis at industrially relevant dimensions remain subjects for future investigation. The performance (expressed as current density at a given cell voltage) of selected perovskite‐based MEAs for PEM electrolysis is shown in Figure 15.

**FIGURE 15:**
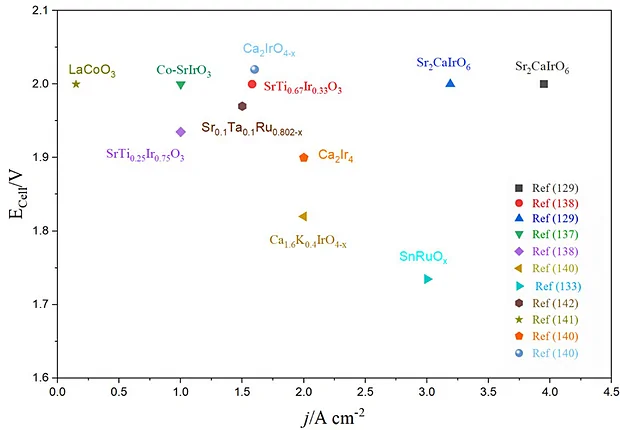
In situ PEM performance up to 2.5 Vcell voltage of selected perovskite‐based catalysts.

## 8 Scale‐Up Strategies

Translating perovskite‐based acidic OER catalysts from laboratory demonstrations to commercially viable PEM water electrolyzers remains a central challenge. Although many perovskite systems exhibit good activity and stability under controlled half‐cell conditions, their practical implementation requires sustained durability at industrial current densities, reproducible large‐scale synthesis, and seamless integration into existing PEMWE architectures [5, 147, 148, 149].

At the materials level, scalability demands synthesis routes that deliver consistent phase purity, controlled stoichiometry, and well‐defined morphology at high production volumes. While laboratory methods such as solid‐state reactions and sol–gel synthesis provide precise compositional control, their limited throughput and energy‐intensive processing restrict industrial applicability. In contrast, scalable approaches such as spray‐flame synthesis, electrospinning, and continuous wet‐chemical routes offer promising pathways for producing nanostructured perovskites with high surface areas and controlled defect chemistries. However, maintaining morphological uniformity, minimizing batch‐to‐batch variability, and preventing undesired phase segregation during scale‐up remain nontrivial challenges [49, 150, 151].

From a device‐integration perspective, compatibility with MEA fabrication processes is equally critical. Scalable perovskite catalysts must exhibit sufficient electronic conductivity, mechanical robustness, and chemical stability within catalyst layers to withstand prolonged operation under acidic conditions. Catalyst–support interactions, ionomer compatibility, and resistance to A‐site leaching or catalyst dissolution must be carefully engineered to prevent performance degradation during long‐term PEMWE operation. Importantly, dynamic surface reconstruction—identified in this review as a key contributor to activity—must be controlled such that it enhances, rather than compromises, mechanical integrity and interfacial stability at scale.

Some of the key catalysts identified, together with their strengths and limitations, are listed in Table 2. It should be noted that the catalytic performance data summarized in Table 2 cannot be directly compared because the reported studies employ different electrolyte compositions and concentrations, including 0.1 M HClO_4_, 0.5 M H_2_SO_4_, 1 M HClO_4_, or O_2_‐saturated electrolytes [56, 57, 74, 83, 84, 94, 95, 97, 107, 114, 119, 128]. Since electrolyte chemistry directly influences catalyst dissolution, surface reconstruction, and the stability of the active surface, differences in activity and durability may not solely originate from the catalyst itself. In particular, HClO_4_ is the predominant electrolyte for fundamental acidic OER investigations, whereas H_2_SO_4_ is employed less frequently because sulfate adsorption may influence the apparent electrocatalytic performance [57, 84, 97]. Moreover, the device‐relevant studies summarized in this review were performed directly in PEM water electrolyzers supplied with deionized water [126, 128, 133]. This emphasizes that catalyst performance obtained in conventional three‐electrode measurements cannot always be directly translated to practical PEMWE operation.

**TABLE 2:** Comparison of Selected Perovskite‐Based OER catalysts (legend: MA = mass activity, η = overpotential, TS = Tafel slope, TOF = Turn over frequency; * = exact value not specified; derived from the graph).

Catalyst system	MA [A g_catalyst_^−1^]	η [mV]	TS [mV dec^−1^]	Stability	TOF [s^−1^]	Dissolution	Catalyst loading [mg cm^−2^]	Electrolyte	Key strengths	Main limitations	Device/scale‐up relevance	Refs.
Sr_3_Ir_2_O_7_	∼160* @300 mV	259 @10 mA cm^−2^	50	Decreased after 1000 polarization cycles	—	—	0.283	0.5 M H_2_SO_4_	High acid stability; layered RP structure; favorable electronic conductivity	Noble‐metal intensive; complex synthesis; limited MEA data	Lab‐scale benchmark; high Ir cost limits scalability	[57]
	∼ 40* @300 mV	296 @10 mA cm^−2^	53	Decreased after 1000 polarization cycles	—	—	0.283	1 M KOH				
Sr_2_IrO_4_	∼ 65* @300 mV	284 @10 mA cm^−2^	57	—	—	—	0.283	0.5 M H_2_SO_4_	Robust layered backbone; stable IrO_6_ framework; tunable electronic structure	Moderate activity; Ir‐rich; limited durability data	Fundamental reference; limited industrial relevance	[57]
	∼ 20* @300 mV	318 @10 mA cm^−2^	63	—	—	—	0.283	1 M KOH				
	—	Slightly higher than overpotential of SrIr_2_O6 303 mV	54.5	—	—	100% Sr leaching after 5 h	0.33	0.1 M HClO_4_				[83]
SrIrO_3_	∼ 20* @300 mV	309 ^56^ 270 ^58^ both @10 mA cm^−2^	61 ^56^ 57.7 ^58^	—	—	—	0.283	0.5 M H_2_SO_4_	High intrinsic activity; metallic conductivity; structural robustness	Sr leaching; noble‐metal dependence; synthesis complexity	Benchmark perovskite; design reference	[57, 84]
	∼ 15* @300 mV	327 @10 mA cm^−2^	70	—	—	—	0.283	1 M KOH				
	—	256 @10 mA cm^−2^	54.5	Less than 28 h @10 mA cm^−^	—	—	0.404	0.1 M HClO_4_				[84]
SrIr_3_O_10_	692.53 @1.5 V	205 @10 mA cm^−2^	66.1	35 h @10 mA cm^−^	1.3 @300 mV	—	0.3	0.1 M HClO_4_	Robust layered backbone; stable IrO_6_ framework;	Ir‐intensive;	Benchmark design reference	[56]
SrIr_2_O_6_	61.3 @1.53 V	303 @10 mA cm^−2^	44.2	300 h @10 mA cm^−^	—	Around 0.5% Sr leaching over 8 h in 1 M HClO_4_	0.33	0.1 HClO_4_	Robust layered backbone; stable IrO_6_ framework;	Ir‐intensive;	Benchmark design reference	[83]
SrIrO‐1100	258 @300 mV	245 @10 mA cm^−2^	47.4	40 h @10 mA cm^−^	—	—	0.404	0.1 M HClO_4_	High activity; enhanced surface reconstruction; bifunctional behavior	Ir‐intensive; reconstruction complicates durability prediction	Mechanistic interest; limited practical deployment	[84]
	225 @300 mV	263 @10 mA cm^−2^	49.7	high catalytic activity after 10 h @10 mA cm^−2^	—	—	0.404	0.5 M H_2_SO_4_				
Y_2_Ru_2_O_7_ (YRO)	269 @300 mV	308 @10 mA cm^−2^	60.7	—	0.13 @300 mV	—	—	O_2_‐saturated 0.5 M H_2_SO_4_	High acidic activity; suppressed dissolution; stable pyrochlore lattice	Ru instability risk; limited compositional tunability	Academic interest; scalability concerns	[94]
Y_1_._7_Sr_0_._3_Ru_2_O_7_ (YSRO‐15)	1018 @300 mV	264 @10 mA cm^−2^	44.8	28 h @10 mA cm^−^	0.27 @300 mV	—	0.071	O_2_‐saturated 0.5 M H_2_SO_4_	Improved electronic structure; enhanced activity; stabilized Ru–O bonding	Noble‐metal usage; limited MEA validation	Academic interest; scalability concerns	[94]
Y_1_._75_Co_0_._25_Ru_2_O_7_−δ (YCR)	See Diagram 4b in ^72^	275 @10 mA cm^−2^	61	3.6% reduction in overpotential @10 mA cm^−2^ after 2000 cycles	—	—	0.2551	O_2_‐saturated 0.5 M H_2_SO_4_	Tuned Ru electronic structure; improved kinetics; cycling stability	Ru dissolution risk; Co stability in acid	Academic interest; scalability concerns	[95]
SrIrO_3_ (Zn‐doped)	102 to 110 @1.55 V	∼ 330* ∼ 350* both @10 mA cm^−2^	>50 <100*	15 to 18 h or 500 to 650 electrochemical cycles	—	—	0.08 (only Ir)	0.1 M HClO_4_	Reduced synthesis energy; reconstruction‐enhanced activity	Noble‐metal content; lifetime prediction uncertain	Potentially scalable; needs MEA testing	[96]
BaNb_0_._2_Ir_0_._8_O_3_ (scratched)	180.476 @1.55 V	250 (300) both @100 A m^−2^	79 (102)	10 h @100 A m^−2^ in 0.5 M H_2_SO_4_ at 25°C instabilities @100 A m^−2^ in 1.7 M H_2_SO_4_ at 40°C	—	< 10 mg L^−1^ Ir and < 10 mg L^−1^ Nb leached after 2 months immersion	0.1 to 0.4	1.7 M H_2_SO_4_	Improved lattice stability; optimized Ir coordination	High Ir content; sulfate sensitivity	Limited device relevance	[97]
HION (Sr_2_IrO_4_‐based)	317 @1.53 V	300 @10 mA cm^−2^	46.9	30 h @10 mA cm^−^	—	∼ 0.15% Ir leaching after 10 h electrocatalysis	0.05	0.1 M HClO_4_	Stable layered backbone; optimized OER energetics	Moderate activity; Ir‐intensive	Fundamental reference system	[114]
Sr_0_._8_K_0_._2_RuO_3_	—	—	59	75% of its initial activity after 20 cycles between 1.2 and 1.5 V at 10 mV s^−1^scan speed	—	—	0.5	0.1 M HClO_4_	Enhanced Ru oxidation state; improved kinetics; structural symmetry	Noble‐metal reliance; limited long‐term data	Lab‐scale; limited practical validation	[107]
S‐doped M‐SrIrO_3_	41.5 @1.55 V	228 @10 mA cm^−2^	58.4	20 h @10 mA cm^−2^	—	—	0.2264	O_2_‐saturated 0.5 M H_2_SO_4_	Improved conductivity; nanosheet morphology; defect‐assisted activity	Ir‐based; moderate durability	Mechanistic insight; early‐stage	[74]
Ir‐STO (SrTi_0_._67_Ir_0_._33_O_3_)	820 @1.525 V	295 @43 mA cm^−2^	43	10 h @10 mA cm^−2^ 10 h @30 mA cm^−2^	—	—	0.21	0.1 M HClO_4_ with N_2_‐bubbling	Reduced Ir content; facet‐dependent activity; good stability	Noble metal still required; limited MEA data	Promising low‐Ir platform	[119]
IrO_x_@LCFN‐96	2316 @1.53 V	120 @10 mA cm^−2^	78.7	20 h @10 mA cm^−2^	—	—	1.1 (catalyst) 0.079 (Ir)	1 M KOH	Crystalline–amorphous synergy; low overpotential; LOM contribution	Composite complexity; noble‐metal content	Lab‐scale; structural complexity limits scale‐up	[121]
	886 @1.5 V	143 @10 mA cm^−2^	93.2	20 h @10 mA cm^−2^	—	—	1.1 (catalyst) 0.079 (Ir)	0.1 M H_2_SO_4_				
	817 @1.83 V	400 @10 mA cm^−2^	192.8	20 h @10 mA cm^−2^	—	—	1.1 (catalyst) 0.079 (Ir)	0.01 M PBS				
SrIrO_3_ nanofibers	—	290 @10 mA cm^−2^	31.6	20 000 s (5.56 h) @10 mA cm^−2^	—	∼ 35%* Sr dissolution after 20 000 s stability test	0.08	1 M KOH	High surface area; efficient charge transport; stable perovskite phase	Ir‐intensive; electrospinning scalability limits	Structured electrodes; niche scalability	[121]
	—	280 @10 mA cm^−2^	44.2	20 000 s (5.56 h) @10 mA cm^−2^	—	∼ 90%* Sr dissolution after 20 000 s stability test	0.08	0.5 M H_2_SO_4_				
	—	300 @10 mA cm^−2^	51.7	20 000 s (5.56 h) @10 mA cm^−2^	—	∼ 50% Sr dissolution after 20 000 s stability test	0.08	1 M PBS				
IrOx/9R‐BaIrO_3_	168 @1.5 V	230 @10 mA cm^−2^	80	48 h @10 mA cm^−2^	—	—	0.283	0.5 M H_2_SO_4_	High Ir mass activity; dynamic surface reconstruction	Noble‐metal heavy; Ba leaching	Mechanistic model system	[152]
Ca_2_−xIrO_4_	—	236 @10 mA cm^−2^	—	100 h in 0.5 M H_2_SO_4_ 300 h in 1 M HClO_4_ both @10 mA cm^−2^	—	—	0.283	0.5 M H_2_SO_4_	Reconstruction‐induced activity; electrolyte‐dependent stability	Limited control over amorphization	Fundamental details; limited scale relevance	[98]
Ca_2_Y_0_._2_Ir_0_._8_O_4_	632.6 @1.5 V	213 @10 mA cm^−2^ (stabilized after 20 cycles)	44.5	168 h @10 mA cm^−2^	0.316 (1137.8 h^−1^)	—	0.283	1 M HClO_4_	Low overpotential; long‐term stability; Y‐stabilized framework	Ir‐based; synthesis complexity	Promising durability; needs scale‐up	[36]
Ir‐CCTO	1195 @300 mV	280 @10 mA cm^−2^	44.7	50 h @20 mA cm^−2^	0.330 @300 mV other voltages see diagram 4K in ^77^	—	0.35	0.1 M HClO_4_	Exceptional Ir mass activity; strong metal–support interaction	Limited stability window; complex nanostructure	Conceptual platform; early‐stage	[99]
Sr_2_CaIrO_6_	—	—	67	1000 h @2 A cm^−2^	—	Selective Sr^2+^ and Ca^2+^ leaching under anodic polarization	0.2 (Ir)	PEMWE (Deionized water)	Ultralow Ir loading; reconstruction‐driven activity; MEA validated	Noble‐metal dependence	High device relevance; strong PEMWE candidate	[126]
SrTi_0_._67_Ir_0_._33_O_3_ (MEA)	809 @1.6 V	—	45	2660 h @1 A cm^−2^	—	Soluble Sr and Ti dissolution compromised durability	0.62 (catalyst) 0.17 (Ir)	Deionized water	Ultralow Ir loading; long‐term durability (>2600 h); strong Ir–O–Ti bonding	Ir still required	High industrial relevance	[133]
Sr_2_MIrO_6_ (M = Ca, Zn, Mg)	900 @1.525 V	—	33	450 h @2 A cm^−2^	0.71 @1.5 V	Selective Sr^2+^ and Ca^2+^ leaching under anodic polarization	0.4 (Ir)	PEMWE (Deionized water)	Edge‐sharing IrO_6_ clusters; high‐current operation	Noble‐metal content; compositional sensitivity	High‐current PEMWE relevance	[128]
γ‐SrIrO_3_ (H‐γ‐SIO)	466 @1.5 V	200 @10 mA cm^−2^	60	1018 h @10 mA cm^−2^	—	∼0.01 ppm leached Ir	0.21	1 M HClO_4_	Open framework; protonated structure; good durability	Metastable phase; synthesis control needed	High durability potential; scale‐up challenge	[106]

Economic and environmental considerations further constrain viable scale‐up strategies. Reducing iridium content without sacrificing durability, employing earth‐abundant elements where possible, and adopting energy‐efficient synthesis routes are essential for achieving cost‐effective deployment. In parallel, the use of environmentally benign solvents, recyclable catalyst architectures, and low‐waste manufacturing processes will be increasingly important as PEMWE technologies move toward gigawatt‐scale hydrogen production.

Bridging the gap between fundamental research and industrial implementation of perovskite‐based acidic OER catalysts requires a holistic approach that integrates materials design, scalable synthesis, electrochemical engineering, and system‐level optimization. Progress in this direction will be pivotal for enabling durable, economically viable, and environmentally sustainable green hydrogen production via acidic water electrolysis.

## 9 Conclusion and Outlook

The development of perovskite‐based electrocatalysts for acidic oxygen evolution has progressed from exploratory materials discovery toward a deeper mechanistic understanding. As highlighted throughout this review, the catalytic performance of acidic perovskites cannot be explained solely by their nominal composition or pristine crystal structure. Instead, activity and stability arise from a complex interplay among composition, defect chemistry, electronic structure, reaction pathways, and the dynamic evolution of the catalyst surface under operating conditions.

From this perspective, several priority directions emerge for advancing perovskite‐based acidic OER catalysts toward technological readiness:

- Predictive design of acid‐resistant compositions must move beyond empirical trial‐and‐error approaches. Integrating data‐driven computational screening with operando experimental validation offers a promising route to identify stable compositional spaces, defect chemistries, and reconstruction pathways.
- Engineering of active sites and reaction pathways represents a promising direction. Rational control of oxidation states, oxygen vacancies, and local coordination environments provides opportunities to tailor not only catalytic activity but also the dominant OER mechanism. Extending concepts such as the oxide pathway mechanism (OPM), which enables direct O–O coupling while preserving lattice integrity, to acid‐stable perovskite systems could provide an attractive route toward simultaneously improving activity and durability.
- Morphology control must be addressed systematically, as nanoscale architecture often dictates mass transport, ionomer interaction, and mechanical integrity in catalyst layers. The development of scalable synthesis strategies capable of delivering controlled morphology without sacrificing phase purity or acid stability remains a key challenge.
- Disentangling true active phases requires broader adoption of surface‐sensitive in situ and operando techniques, particularly under device‐relevant conditions. Operando spectroscopic techniques or half‐cell measurements are essential for capturing the evolution of the catalyst surface but, on their own, are far from sufficient to capture the complex chemical, mechanical, and electrochemical environments present in working MEAs. As demonstrated throughout this review, MEA‐level testing is indispensable for identifying realistic degradation modes, reconstruction dynamics, and transport limitations.
- Future catalyst development should increasingly consider multifunctionality rather than activity alone. Rationally engineered perovskite catalyst layers may offer the opportunity not only to provide efficient OER active sites but also to improve electrical contact or interface stability. This raises the possibility for eliminating Pt coatings currently required on porous transport layers to suppress interfacial contact resistance. Such opportunities are rarely apparent from conventional three‐electrode measurements and only emerge through systematic evaluation under realistic MEA conditions.

The studies reviewed here also reveal an important limitation of the current state of the field: the most active and durable acidic perovskite‐structured systems remain predominantly dependent on Ir or other PGMs. In many of these materials, the initial perovskite‐structured surface undergoes substantial reconstruction during OER, ultimately forming IrO_x_‐rich species that constitute the currently existing catalytic phase, similarly to conventional benchmark catalysts. Nevertheless, the perovskite lattice remains fundamentally important, as it governs Ir utilization and tailors the electronic environment of the active phase. More importantly, this materials platform offers opportunities that extend beyond intrinsic catalytic activity. Rational engineering of the perovskite composition and surface chemistry may enable multifunctional catalyst layers of electrodes with improved catalyst–ionomer interactions, optimized wetting behavior, or enhanced water management, thereby facilitating proton access to active sites or increasing catalyst utilization. Such advantages become apparent only under realistic MEA operating conditions and therefore highlight the importance of evaluating perovskite catalysts beyond conventional half‐cell measurements.

Beyond performance optimization, future research must confront issues of scalability, sustainability, and life‐cycle impact. Reducing iridium loading while maintaining durability remains essential, as does the development of synthesis routes compatible with large‐scale manufacturing. At the same time, the use of abundant elements, eco‐friendly solvents, and recyclable catalyst architectures will be necessary to minimize environmental and economic costs.

Ultimately, the transition of perovskite‐based acidic OER catalysts from laboratory studies to operational hydrogen technologies will require evaluation frameworks that extend beyond short‐term activity metrics. Testing of these materials must therefore rely on commonly agreed, harmonized and standardized durability tests or accelerated stress protocols to allow meaningful comparison with the high‐performing traditional OER catalysts. If the inherently dynamic nature of perovskite catalysts is embraced and materials design is aligned with device‐level requirements from the outset, perovskite‐based systems  are poised to play a central role in enabling durable, scalable, and economically viable next‐generation water electrolyzers.

## Author Contributions

**Jala Bib Khan**: conceptualization (supporting); data curation (lead); formal analysis (lead); visualization (lead); writing – original draft (lead). **Norbert Kazamer**: conceptualization (lead); formal analysis (supporting); validation (lead); writing – original draft (supporting); project administration (lead). **Marco Brand**: validation (supporting); visualization (supporting); writing – review and editing (supporting); **Tim Hülser**: validation (equal); writing – review and editing (supporting); project administration (lead). **Clemens Pollerberg**: validation (supporting); writing – review and editing (supporting). **Ulrich Rost**: validation (equal); writing – review and editing (supporting).

## Conflicts of Interest

The authors declare no conflict of interest.

## Data Availability

Data sharing not applicable to this article as no datasets were generated or analysed during the current study.
